# Connectivity gradients on tractography data: Pipeline and example applications

**DOI:** 10.1002/hbm.25623

**Published:** 2021-09-24

**Authors:** Guilherme Blazquez Freches, Koen V. Haak, Christian F. Beckmann, Rogier B. Mars

**Affiliations:** ^1^ Donders Institute for Brain, Cognition and Behaviour, Radboud University Medical Center Nijmegen The Netherlands; ^2^ Donders Institute for Brain, Cognition and Behaviour, Radboud University Nijmegen The Netherlands; ^3^ Wellcome Centre for Integrative Neuroimaging, Centre for Functional MRI of the Brain (FMRIB), Nufeld Department of Clinical Neurosciences John Radclife Hospital, University of Oxford Oxford UK

**Keywords:** connectivity, gradients, topography, tractography

## Abstract

Gray matter connectivity can be described in terms of its topographical organization, but the differential role of white matter connections underlying that organization is often unknown. In this study, we propose a method for unveiling principles of organization of both gray and white matter based on white matter connectivity as assessed using diffusion magnetic ressonance imaging (MRI) tractography with spectral embedding gradient mapping. A key feature of the proposed approach is its capacity to project the individual connectivity gradients it reveals back onto its input data in the form of projection images, allowing one to assess the contributions of specific white matter tracts to the observed gradients. We demonstrate the ability of our proposed pipeline to identify connectivity gradients in prefrontal and occipital gray matter. Finally, leveraging the use of tractography, we demonstrate that it is possible to observe gradients within the white matter bundles themselves. Together, the proposed framework presents a generalized way to assess both the topographical organization of structural brain connectivity and the anatomical features driving it.

## Graphical abstract

In this work, we introduce a pipeline for deriving overlapping connectivity gradients on gray and white matter, according to their white matter connections. We then use the pipeline to derive connectivity gradients on the prefrontal and occipital cortices as well as the optic radiation. Finally, we backproject these gradients onto the input white matter, revealing their origins.

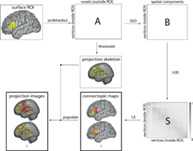

## 1 INTRODUCTION

The function of a given brain region is defined by its internal aspects—cytoarchitectonical and cytochemical features but also by the afferent and efferent projections it has from and to other parts of the brain, respectively (Mars, Passingham, & Jbabdi, 2018; Passingham, Stephan, & Kötter, 2002; Saygin et al., 2016). More specifically, these connectivity patterns give rise to networks and the dynamic balance between these networks characterizes function and, consequently, behavior (Jbabdi, Sotiropoulos, & Behrens, 2013; Peer, Nitzan, Bick, Levin, & Arzy, 2017).

While most descriptions of the connections between brain areas have focused on a region‐to‐region map, it is increasingly apparent that there is useful information in the topographic organization of connections within and across such regions (Haak & Beckmann, 2020; Jbabdi et al., 2013). For instance, regions organized in a rostral–caudal fashion in the frontal lobe connect to regions in a caudal–rostral fashion in the parietal lobule, mirroring this principle of organization across different parts of the brain (Vijayakumar et al., 2018). Most sensory networks also rely on topographically disposed connections being conserved from the sensory input site all the way to the cortex, allowing them to maintain effectiveness in case of a lesion in the pathway (Kaas, 1997). Global gradients also exist across species and reflect a hierarchies in the cognitive landscape, with multimodal regions corresponding to hubs of the default‐mode network sitting at one extreme and primary sensory regions such as primary visual area V1 being at the other (Margulies et al., 2016; Xu et al., 2020).

Yet, despite the expanding body of evidence pointing toward principles of topographic organization and preservation of connections, the overwhelming majority of models for distributed processing are predicated on the notion of homogeneous, piecewise constant connection signatures within discrete brain regions (Eichert et al., 2018). Topographic organizational principles of connectivity are difficult to establish with most current parcellation techniques that aim to separate the brain into distinct parcels based on their shared within‐area connectivity and distinct between‐area connectivity profiles (Eickhoff et al., 2015; Klein et al., 2007; Neubert, Mars, Thomas, Sallet, & Rushworth, 2014), and thereby ignore fundamental principles of topographically organized heterogeneity within areas (Haak & Beckmann, 2020). Further, this difficulty is exacerbated by the possible presence of connectional multiplicity, that is, the presence of multiple overlapping connection topographies caused by differential spatial patterns of all afferent projections (Haak & Beckmann, 2020; Haak, Marquand, & Beckmann, 2017). These allow for the computation of complex functions using relatively simple spatial rules and their disentanglement may provide important primers for computational models of high order brain functions (Jbabdi et al., 2013).

From a computational standpoint, the topographic disposition of connections can increase the efficiency of communication between regions since neurons that are more likely to interact are situated closer together which, in turn, can reduce wiring costs. This type of organization has now been shown to not only be present in many parts of the brain and across vertebrates—suggesting that there is an evolutionary advantage to it—but also to have functional relevance for behavior (Marquand, Haak, & Beckmann, 2017; Tinsley, 2009). The topographic regularity of the connections themselves has also seen a recent surge of interest, with studies showing that the axons connecting topographical maps conserve the same spatial pattern along their entire trajectory (Aydogan & Shi, 2016, 2018; Wang, Aydogan, Varma, Toga, & Shi, 2018).

Haak et al. (2017) recently proposed a method aimed at quantifying topographic patterns by finding the underlying, dominant directions of connectivity change within a brain region. This method was based on earlier work using Laplacian Eigenmaps (LE) proposed by Cerliani et al. (2012). Focusing on resting state fMRI, Haak and colleagues presented a pipeline for revealing these connectopic topographies—connectopies—and, importantly, provided a principled statistical framework for comparing connectopies from different subjects and to test for associations with secondary measurements such as demographics and behavior. This approach has been successful in demonstrating connectopic organization in the primary visual and motor cortices, but also in revealing behaviorally relevant topographies in the striatum (Marquand et al., 2017), across the hippocampus (Przeździk, Faber, Fernández, Beckmann, & Haak, 2019; Vos de Wael et al., 2018), entorhinal cortex (Navarro Schröder, Haak, Zaragoza Jimenez, Beckmann, & Doeller, 2015) insula (Tian & Zalesky, 2018), and the anterior temporal lobe (Faber, Przeździk, Fernández, Haak, & Beckmann, 2020).

While resting state MRI‐based functional connectivity gradients capture subject‐specific, biologically relevant information, they rely on BOLD signal suffering from the same limitations as any rsfMRI connectivity measures such as a high dependence of subject state (e.g., eyes open vs. eyes closed; Cole et al., 2010). Furthermore, if connectivity gradients are to be used as the basis for biology‐based models of brain function, the physical implementation and evolution of these gradients must be investigated in addition to their functional consequences. Moreover, focusing on functional activation precludes the application of this technique in ex‐vivo samples, which would open up the possibility to use this framework in samples that can be directly validated against histological investigations and comparative studies using post‐mortem tissues. Finally, as structural connectivity is constituted by a discrete set of common elements (white matter tracts), we are able to back‐project the gradients onto our input space, revealing its contribution to the observed graded connectivity pattern changes. As such, it is crucial to map gradients in structural brain connectivity in order to resolve how they have been driving the observed functional heterogeneity and multiplicity. Here, we demonstrate both principles using diffusion MRI data, opening up the way to a better understanding of this new way to understand the physical architecture underlying neural computations.

**FIGURE 1:**
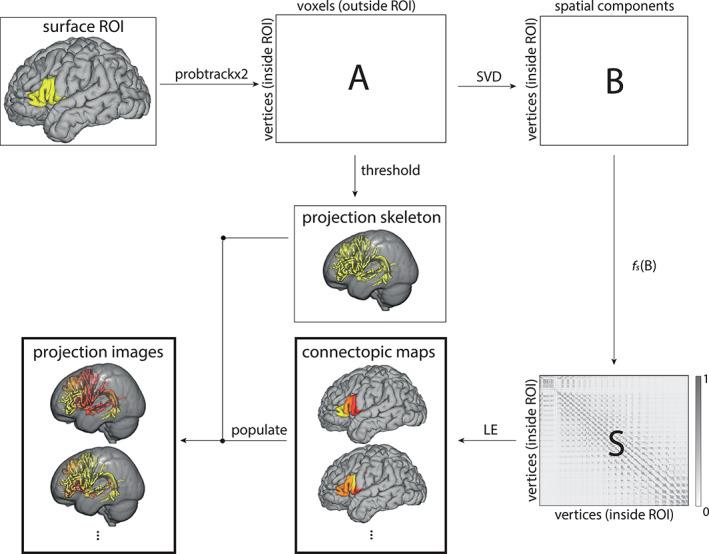
Schematic overview of the proposed connectopic‐mapping framework. Probabilistic tractography is run from either a surface or a volume seed region. The resulting connectivity matrix (A) go through dimensionality reduction via Singular Value Decomposition (SVD), resulting in matrix B. Matrix B is then transformed into a similarity matrix (S), which is used to compute the similarity graph. Finally, the graph's Laplacian is decomposed into its eigenvectors that correspond to the connectopic maps of the seed region. Their corresponding projections in target space (projection images) are then computed by populating the thresholded connectivity matrix with the values coming from the corresponding connectopic maps. We discuss each step in more detail in Section 2

This article shows that white matter pathways exhibit multiple overlapping, topographically organized modes of connectivity. We outline a technique (a schematic can be found in Figure 1) based on the connectopic mapping approach introduced in Haak et al. (2017) for resting‐state functional connectivity, but adopted to use in the context of diffusion weighted imaging (DWI) data and probabilistic tractography for generating gradually changing white matter connectivity estimates. This technique extends previous applications of the LE approach on DWI data (Bajada et al., 2017; Cerliani et al., 2012) by characterizing the modes of change within the white‐matter tracts themselves rather than their projections onto the cortical surface, and was recently used to uncover three overlapping modes of connectivity in the temporal lobe, associated these modes with specific white matter contributions and assigned them as principles of functional organization of the temporal lobe (Blazquez Freches et al., 2020). Here, we show that these modes of structural connectivity exhibit high levels of reproducibility, recapitulate known anatomical boundaries between cortical regions and tract subdivisions, and represent different features of the underlying white matter connectome. Specifically, and through application in model systems (language and vision), we show that the underlying white matter tracts contribute differently across systems, hemispheres and modes of connectivity. Additionally, we demonstrate through an example tract (optic radiation) that gradual connectivity changes are conserved along the connecting white matter fibers themselves, adding a different layer of complexity to the study of connectivity topographies (connectopies).

## 2 METHODS

All analysis was performed on both Sessions 1 and 2 of the test–retest cohort in the Human Connectome Project (HCP; Van Essen et al., 2013) dataset. A different subset of subjects from this dataset was used for direct comparison with an earlier study (Jakobsen et al., 2016). Numerical results are reported for both sessions and figures refer to results from Session 1 unless indicated otherwise. The corresponding figures for Session 2 can be found in the Supporting Information figures section.

### 2.1 Data

Forty‐four subjects (28 females, 4 left handed, aged 22–35 years) of the HCP test–retest cohort were selected. These subjects were scanned in two different sessions (referred to as Session 1 and Session 2). A second subset of the HCP dataset was selected to match the one studied by Jakobsen et al. for direct comparison of results. (Jakobsen et al., 2016; 101 subjects, 59 females, aged 22–35 years). Within both subsets of subjects, no subsequent preprocessing steps were employed other than the ones already performed by the HCP minimal preprocessing pipeline (Glasser et al., 2013; Sotiropoulos et al., 2013). Importantly, posterior distributions of fiber orientations for probabilistic tractography were generated using FMRIB software library (FSL's) BEDPOSTX (Behrens, Berg, Jbabdi, Rushworth, & Woolrich, 2007; Jbabdi, Sotiropoulos, Savio, Graña, & Behrens, 2012). The data spatial resolution was 1.25 mm isotropic.

### 2.2 Pipeline summary

The proposed pipeline is illustrated in Figure 1. Probabilistic tractography is run from seed region to the rest of the hemisphere (for simplicity a stop mask was put at the midsagittal section in order to ignore inter hemispheric crossings—these may continue tracking after reaching their tract endpoint, inducing spurious connections). The resulting connectivity matrix is then transformed into a similarity matrix, that is used to compute the adjacency graph (in our case, we computed the minimum number of neighbors needed to make a connected graph). Finally, the graph's Laplacian is decomposed into its eigenvectors that correspond to the connectopic maps of the seed region. Their corresponding projections in target space (projection images) are then computed by populating the thresholded connectivity matrix with the values coming from the corresponding connectopic maps.

### 2.3 Region of interest selection—surfaces

Group region of interest (ROI)'s for each hemispheres were created by defining a 95% consensus mask across all subjects' Brodmann maps (Fischl et al., 2008). The consensus mask would contain a specific vertex if that vertex were assigned to the corresponding Brodmann area in at least 84 of the 88 data points (44 subjects scanned twice). For our study, two regions were selected: Brodmann areas 44 and 45 merged (BA 44/45—association cortex) and 17 (V1—primary cortex). These two regions were analyzed in both hemispheres. An identical approach was followed in order to create 50% agreement and 1 subject agreement (where the consensus mask contained all the vertices that were classified as belonging to Brodmann area 44/45 in half the subjects of at least in one subject respectively) masks for Brodmann areas 44/45. Finally, in 101 subjects of the full HCP cohort (of which six belonged to the 44 test–retest cohort taken originally), manually delineated BA 44/45 masks (Jakobsen et al., 2016) on the left hemisphere were used. In FSL, these surfaces were used directly as the seeds for tractography, by transforming them from surface space (in mm) to voxel space (in voxel coordinates) using the caret convention.

### 2.4 Region of interest selection—volume

Individualized white matter tract thresholded tractograms were obtained using FSL's XTRACT (Warrington et al., 2020) following the procedure described in (Mars et al., 2018). In our study, the optic radiation in both hemispheres was selected. These individualized tracts were then intersected with a coronal plane at *y* = −58.5 (where all subjects had voxels pertaining to the optic radiation) in MNI space to produce individual optic radiation cross sections.

### 2.5 Probabilistic tractography

The first step in the pipeline was to run probabilistic tractography seeding from the chosen ROI at the individual mid‐thickness surface level towards the whole hemisphere (when seeding from the surface) or from the volumetric cross section to the rest of the tract (when seeding from a tract cross section in volume space). Surface seeds were warped to volume space and removed from the target hemisphere so that self‐connectivity effects would be mitigated. Stop masks were placed at the pial surface and at the mid‐sagittal plane so that streamlines would not leave the brain or cross hemispheres. FSL's PROBTRACKX was used with the following settings: 10,000 streamlines per voxel, maximum path length of 2,000 steps, step size of 0.5 mm, and the “matrix2” mode (thus saving the result of the probabilistic tractography in a connectivity matrix corresponding to the visitation counts of every seed voxel to each target voxel). This yielded a *seed × hemisphere* matrix that corresponds to A in Figure 1.

### 2.6 Dimensionality reduction

To reduce computation, matrix A's dimensionality was reduced using SVD resulting in Matrix B (Figure 1) describing the connectivity fingerprint of each vertex in the seed which each of a set of spatially uncorrelated components. Matrix B is thus of size *seed × components*.

### 2.7 Similarity matrix

To compute the between‐vertex similarity between seed vertices, a similarity function was applied to matrix B. In this pipeline, the *η* ^2^ coefficient was chosen (Cohen et al., 2008). This coefficient expresses similarity between connectivity fingerprints by how much explained variance one accounts on the other with the following formula $\eta^{2}=\frac{{\mathrm{SS}}_{\text{fingerprint}}}{{\mathrm{SS}}_{\text{total}}}$ where SS_fingerprint_ represents how much variance of the fingerprint being compared is explained by the target fingerprint and SS_total_ represents the total variance in the fingerprint under comparison. The result of this step is matrix S (Figure 1) of size *seed × seed*. The values in matrix S range from 0 (completely dissimilar) to 1 (equal).

### 2.8 Graph construction

The similarity matrix S was transformed into a weighted graph by means of a *k*‐nearest neighbors approach with the number of neighbors being the minimum necessary so that the resulting graph only contained one connected component.

### 2.9 Dimensionality estimation

A dimensionality estimation approach was used to limit our group analysis to the minimum common number of dimensions across all subjects. This was done by estimating the dimensionality of each individual network graph using Maximum Likelihood Estimation (MLE) of intrinsic dimensionality (Levina & Bickel, 2004) and choosing the minimum common subset across subjects. In all cases, the common number of estimated dimensions was 2.

### 2.10 Laplacian Eigenmaps

The selected regions' LE was obtained by performing the generalized Eigen decomposition of the graph Laplacian, after discarding the first eigenvector (0‐valued eigenvalue; (von Luxburg, 2007). In this study, the two eigenvectors (normalized between 1 and 10) associated with first two nonzero eigenvalues were investigated (These eigenvectors are referred throughout this article as connectopic topographies or “connectopies.” When overlaid on an anatomical image, the connectopic topographies are referred to as connectopic maps. Group‐level connectopic maps were obtained by averaging all subjects' connectopic maps within a dataset (test or retest). Averaging subjects' matrix2 incoming from FSL's PROBTRACKX was not possible since the self‐connectivity exclusion masks were slightly different from subject to subject.

### 2.11 Projection images

To investigate how the connectopic topographies for a given gray matter area are related to connections with underlying white matter, we created tract projection images. First, we created a projection skeleton in volume space, showing for each voxel in the target hemisphere how often a streamline from seed had reached it. This projection skeleton was created by thresholding (such that only voxels visited by at least 1% of streamlines or a given seed vertex were considered) and binarizing matrix A. Second, we populated each voxel of the projection skeleton with the weighted average connectopy value of the top three vertices which streamlines hit that target voxel the most, thereby producing the projection images. Each connectopic map will have one projection image associated with it. Figure 1 shows one example of a projection image.

### 2.12 Tract skeletons

To identify the tracts that contributed to the observed connectopies, we next determined which parts of the projection images were composed of specific white matter tracts. In order to achieve this, every projection skeleton was multiplied with individualized white matter tract thresholded tractograms obtained using FSL's XTRACT (Warrington et al., 2020) following the procedure described in (Mars, Sotiropoulos, et al., 2018). We refer to the resulting images as tract skeletons.

### 2.13 Tract projections

**FIGURE 2:**
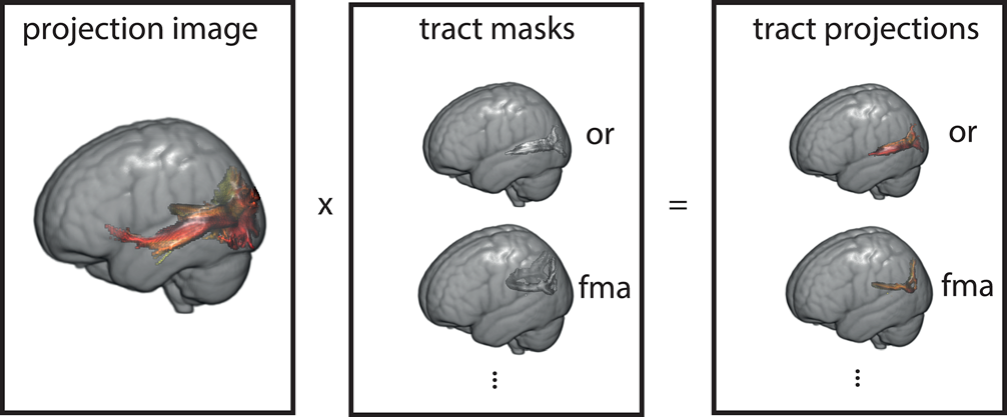
Illustration of the creation of tract projections. A representative projection image (first connectopy) is multiplied with 39 individualized white matter tract masks obtained with FSL's autoPTX to create tract projections. FMA, forceps major; OR, optic radiation

In order to separate the contributions of each white matter tract to the overall connectome, every projection image was multiplied with the previously obtained individualized white matter tracts. We refer to the resulting images as tract projections. The process is illustrated in Figure 2. For clarity, a given subject for which two connectopic maps are calculated will have two projection images and 78 tract projections (one for each tract*connectopic map combination).

### 2.14 BA44/45 parcellation

Brodmann areas 44 and 45 were merged in one single ROI for this study. To investigate if the first connectopy of BA 44/45 could parcellate this single ROI into its two separate components reliably, the *k*‐means++ algorithm (Arthur & Vassilvitskii, 2007) was used (with two centroids) to create predicted BA44 and BA45 masks that served as a target for validation. The resulting predicted masks were compared with the initial BA 44 and BA 45 masks using the Dice coefficient (DC; Dice, 1945), where $\mathrm{DC}=\frac{2\left|X\cap Y\right|}{\left|X\right|+\left|Y\right|}$; where |*X*| and |*Y*| represent the number of elements in each set and X∩Y represents the common elements for both sets. Dice similarity results were defined as the bootstrapped 95% confidence interval of the mean result and the bootstrap was made with 10,000 samples. Additionally, and in order to evaluate the influence of the presence of outliers on the ROI, the 50 and 1% agreement BA 44/45 masks were subjected to the same procedure. Finally, and to compare the accuracy of the parcellation between manual and algorithm made labels, manually delineated and merged BA 44/45 (left hemisphere only) masks from (Jakobsen et al., 2016) were also analyzed in a similar fashion. In this last case, the DC was calculated between the intersection of the manual masks and the 95% agreement BA 44/45 predicted masks as two avoid lower scores caused by mismatches of the initial masks.

### 2.15 Cross‐subject and cross‐session reproducibility

In order to assess cross‐subject and cross‐session reliability of the connectopic maps (the procedure was the same for cortical seeds and white matter seeds), the intra‐class correlation coefficient case 2.1 (Shrout & Fleiss, 1979) with *k* = 2 for both cross‐subject and cross‐session ICC where $\mathrm{ICC}=\frac{\left(\mathrm{BMS}-\mathrm{EMS}\right)}{\mathrm{BMS}+\left(k-1\right)\mathrm{EMS}+\frac{k\left(\mathrm{JMS}-\mathrm{EMS}\right)}{n}}$, where *n* is the number of “targets” (here voxels in our ROI), BMS is the between targets mean square, EMS is the error mean square, and JMS is the between “judges” mean square (here sessions or subjects). Both cross‐subject ICC and cross‐session ICC were defined as the bootstrapped 95% confidence interval of the means of their respective definitions. The bootstrap was made with 10,000 samples.

### 2.16 Mate‐based retrieval rate

To further investigate the reliability and uniqueness of connectopic maps, a mate‐based retrieval rate experiment was performed. An exact matching criterion was employed meaning that a match was considered successful if the connectopy based on the first session run attained maximal correlation with the corresponding connectopy in the second session. The matching accuracy was then the sum of matches divided by the total number of subjects. This approached was used both in surface seeds (BA 44/45 and V1) and white matter (OR cross‐section).

### 2.17 Projection skeleton—Lateralization

Tract skeletons (39 white matter individual tract masks multiplied by the projection skeletons) were compared in terms of lateralization index (Thiebaut de Schotten et al., 2011). Lateralization index was defined as: $\frac{P_{\text{right}}-P_{\text{left}}}{P_{\text{right}}+P_{\text{left}}}$, where *P* is the proportion of volume of a given tract to the full projection image volume. For any ROI considered, only tracts that represented at least 3% of the total projection image were considered and kept for tract projection. Every tracts' lateralization index was classified as left lateralized, right lateralized or non‐lateralized as the result of a one sample *t*‐test (Bonferroni corrected) across all subjects on each session.

### 2.18 Projection image—Tract projection

To establish whether the information present in projection images was sufficient for separating the contributions of different white matter tracts in different connectopies, the bootstrapped 95% confidence interval of the mean value was calculated in each relevant tract projection after multiplication with a white matter mask to minimize effects from gyral biases and cortical terminations in every subjects' projection images.

### 2.19 Tract cross‐sectional gradient—Gradient profiles

As every individual had slightly different optic radiation tract intersection coordinates, at *y* = −58.5, an additional step towards normalizing the *z* coordinate was made. In every subject, the range of values along the *z* coordinate was upsampled using linear interpolation to 100 data points between 0 (most ventral coordinate for a given subject) and 1 (most dorsal coordinate for that given subject). Finally, every subject's gradient profile along the *z* axis was calculated by averaging all values along the *x* direction (medial to lateral) for every normalized *z* coordinate. This data manipulation produced an array of dimensions 1 × 100 (interpolated size of the *z* dimension) for every subject, representing the average projection image value at *y* = 58.5 for any normalized *z* coordinate.

### 2.20 Tract cross‐sectional gradient—Projection image profiles

Projection image values on g1 were calculated by normalizing the *z* coordinates in the same fashion as had been done with gradient images. An additional step was made, which was to average along the y‐coordinate from the posterior end of the projection images up to *y* = −58.5. Thus, in this case, each individual 1 × 100 array represents the average projection image value from *y* = −58.5 until its most posterior point, at every normalized *z*‐coordinate point.

The projection image values on g2 were obtained similarly, by only switching dimensions *y* and *z*. Values were averaged across the *z*‐coordinate and normalized in the *y*‐axis.

## 3 RESULTS

We investigated the potential of the connectopic mapping approach to unravel overlapping modes of brain organization using diffusion MRI tractography data. First, we demonstrate the method's ability to find overlapping modes of organization of brain areas based on their long‐range connectivity and validate the robustness of the results across subjects and sessions. Second, we show that these principles recapitulate and characterize organizational principles shown with other methods while further giving insights on their origin. Finally, we demonstrate how the method can elucidate the organization of the white matter itself, demonstrating its potential in particular in tractography data. All tables represent data from both cohorts. Figures represent data from the test cohort, with the corresponding retest cohort being represented in Supporting Information section.

### 3.1 Connectopic mapping show biologically meaningful maps at a group level

Group connectopic maps were created in order to unravel the global common modes of connectivity of selected cortical regions as follows. Tractography was performed from selected ROIs toward the whole hemisphere and the resulting individual tractograms were submitted to the connectivity gradient pipeline of Figure 1. Finally, the connectopic maps were averaged across subjects. In both case studies, our pipeline recommended the analysis of the first two dimensions of the data, from herein referred as dominant mode and second dominant mode of connectivity. This dimensionality estimation was performed using MLE (Levina & Bickel, 2004).

**FIGURE 3:**
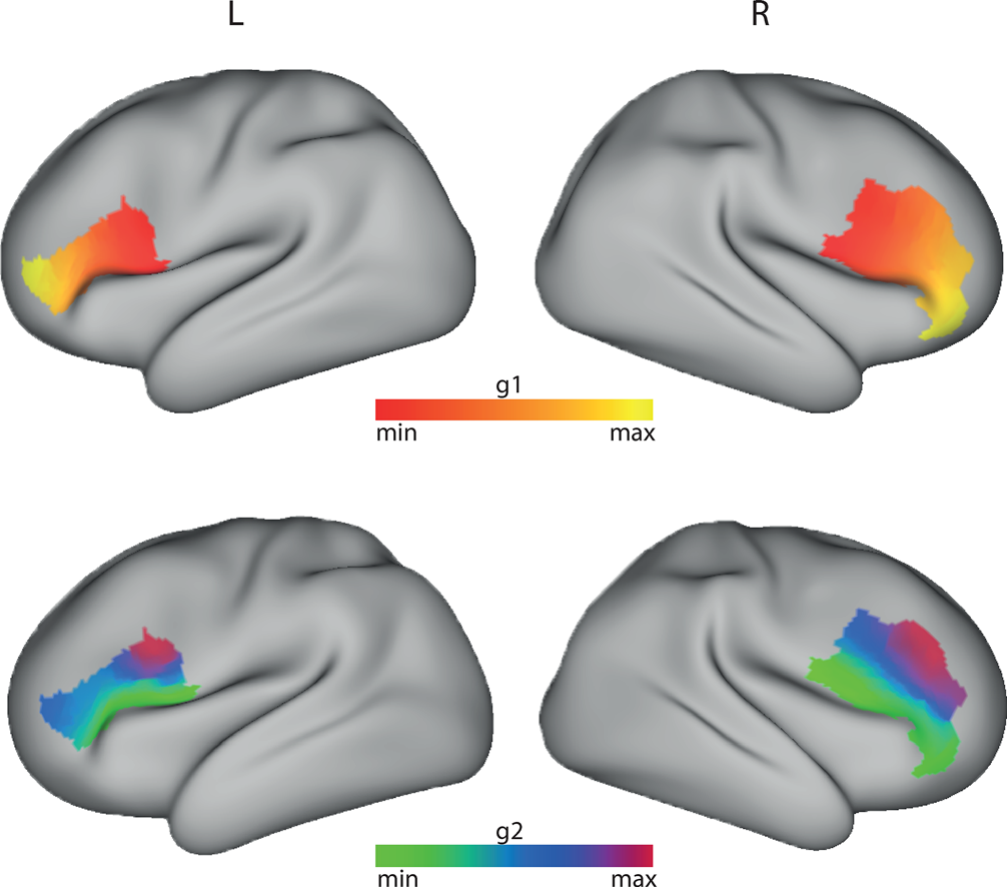
Group connectopic maps of BA 44/45 overlaid on an inflated cortical surface (test cohort). The top row shows the connectopic maps for the dominant connectivity mode (g1). The bottom row shows the connectopic for the second dominant connectivity mode (g2). The L R labels refer to the left and right hemisphere, respectively

In the case of Brodmann's areas 44/45, we found that the dominant mode of connectivity (Figure 3—top row) showed a bilateral anterior–posterior gradient consistent with the anterior–posterior division between BA44 and BA45 as previously demonstrated using connectivity‐based parcellations (Anwander, Tittgemeyer, von Cramon, Friederici, & Knosche, 2006; Friederici, 2009; Glasser & Rilling, 2008) and their functional segregation (Friederici et al., 2013; Hagoort, 2013; Jakobsen et al., 2016).

The second dominant mode of connectivity (Figure 3—bottom row) revealed a ventral‐dorsal connectivity gradient consistent with the dual pathway model for language processing (Friederici et al., 2013; Hickok & Poeppel, 2007; Saur et al., 2008). Taken together, these results suggest that, as rsfMRI, DWI‐based connectopic mapping is able to disentangle biologically meaningful, overlapping modes of connectivity present within a ROI.

**FIGURE 4:**
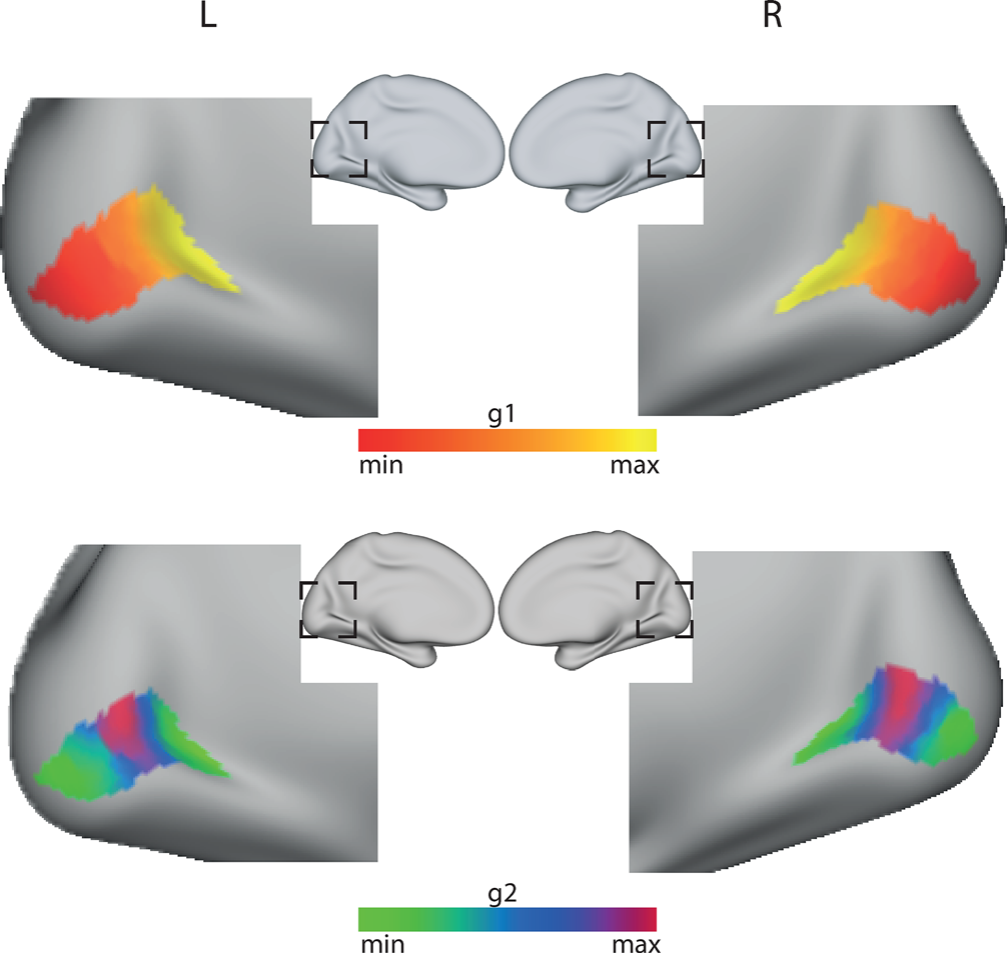
Group connectopic maps of V1 (occipital pole plane‐dashed line) overlaid on an inflated cortical surface (test cohort). The top row shows the connectopic maps for the dominant connectivity mode (g1). The bottom row shows the connectopic for the second dominant connectivity mode (g2)—deemed unreliable by the dimensionality estimation algorithm. The L R labels refer to the left and right hemisphere, respectively

Likewise, Brodmann area 17 (referred as V1), has been shown to map eccentricity in the visual field along the calcarine sulcus from the posterior to the anterior parts (Dougherty et al., 2003; Wandell, Dumoulin, & Brewer, 2007). More specifically, the posterior end of this mapping is assigned to the fovea, giving large numbers of neurons the task of processing information from this small region of the visual field and thus enabling the fine spatial resolution near the center of the visual field (Azzopardi & Cowey, 1993; Daniel & Whitteridge, 1961; Duncan & Boynton, 2003). The group dominant connectivity mode of V1 (Figure 4—top row) presented a similar posterior–anterior gradient, in agreement with the previous study by Haak et al. (2017) using resting state fMRI instead of tractography. In contrast to the fMRI results of the previously study, however, the MLE dimensionality estimator did not indicate evidence of more than one gradient in the diffusion data. Indeed, the second dominant connectopy (Figure 4—bottom row) showed a radial component that did not match previous functional mappings found in this region of the cortex.

### 3.2 Individual level connectivity gradients capture subject‐specific information

The method's capacity for mapping connectivity gradients at the individual level was accessed by analyzing the same connectivity gradients of BA44/45 and V1 at the individual subject level. We used 44 participants for whom both test and retest data were available, meaning that for every participant we had two sets of results—one for each session. The robustness of these maps was evaluated across sessions and across subjects using the intra‐class correlation coefficient—ICC (case 2,1); see Section 2 (Shrout & Fleiss, 1979). Results are summarized in Table 1.

**TABLE 1:** Reproducibility of connectopic mapping at the single‐subject level

ICC	BA 44/45 (g1)	BA 44/45 (g2)	V1 (g1)	V1 (g2)
Between sessions	L—0.918 [0.884–0.936]	L—0.651 [0.522–0.736]	L—0.863 [0.831–0.895]	L—0.581 [0.463–0.657]
	R—0.769 [0.670–0.837]	R—0.602 [0.454–0.703]	R—0.840 [0.756–0.880]	R—0.553 [0.462–0.627]
Between subjects				
Session 1	L—0.810 [0.801–0.817]	L—0.423 [0.405–0.451]	L—0.844 [0.836–0.850]	L—0.482 [0.461–0.502]
	R—0.618 [0.599–0.634]	R—0.470 [0.444–0.494]	R—0.825 [0.815–0.833]	R—0.550 [0.534–0.564]
Session 2	L—0.813 [0.803–0.822]	L—0.383 [0.356–0.402]	L—0.800 [0.792–0.809]	L—0.520 [0.507–0.535]
	R—0.611 [0.594–0.626]	R—0.479 [0.455–0.502]	R—0.746 [0.731–0.761]	R—0.451 [0.433–0.466]

*Note*: Results are compared between sessions from the same subject in both sessions and between pairs of subjects within a session. Reported values represent the average intra‐class correlation coefficient across same subject pairs in different sessions (between sessions) or different subject pairs in the same session (between subjects). Values between square brackets indicate the lower and upper bounds of the bootstrapped 95% confidence interval with 10,000 samples, respectively.

Abbreviations: L, left hemisphere gradient; R, right hemisphere gradient.

For both in the BA 44/45 and V1 masks, the reproducibility between sessions and between subjects decreases as we move from the first gradient—dominant mode of connectivity (g1) to the second gradient ‐ second dominant mode of connectivity (g2). This indicates that dominant connectivity modes are more similar across subjects (between subjects ICC) and more robust (between sessions ICC). The between subjects ICC is always lower (in either session) than the between session ICC, indicating that individual connectopies retain subject‐specific information.

Interestingly, we observed an asymmetry effect on the first gradient (g1) of BA 44/45 whereas in V1 there is symmetry across gradients for the between subjects and between sessions ICC. In the principal gradient of BA 44/45, the ICC is significantly higher in the left hemisphere for both between session and between subjects ICC. This indicates that the dominant connectivity mode of BA 44/45 is both more reproducible and more homogenous between subjects in the left hemisphere. This asymmetry effect disappears when the second dominant connectivity mode is analyzed.

On the basis of observing that both V1 and BA 44/45 had a high between session ICC indicating the robustness of their respective dominant connectivity modes, we hypothesized that despite these similar results, the dominant gradients of BA44/45 would be more subject‐specific than the dominant gradients of V1. To test this hypothesis, we performed a mate‐based retrieval rate analysis. For each connectivity mode, we computed how often the connectivity mode of a given subject based on the first session attained maximal correlation with its corresponding connectivity mode in the second session (compared to all others). The results are displayed in Table 2.

**TABLE 2:** Mate‐based retrieval rate for first and second dominant connectopies for BA 44/45 and V1

Mate‐based retrieval rate	BA 44/45 (g1)	BA 44/45 (g2)	V1 (g1)	V1 (g2)
Between sessions	L—59.1% (65.9%)	L—36.4% (50%)	L—13.6% (31.8%)	L—22.7% (34.1%)
	R—43.2% (52.3%)	R—38.6% (43.2%)	R—15.9% (25%)	R—20.5% (29.5%)

*Note*: Reported values represent the percentage of subjects, for which the connectopy in one session was maximally correlated to the corresponding connectopy in the other session. Values in brackets represents the same measure, but allowing for the correspondent connectopy to be in the top three matches.

Abbreviations: L, left hemisphere; R, right hemisphere.

The first and second gradients in both BA 44/45 (15–25 times above chance level) and V1 (5–9 times above chance level) had a mate‐based retrieval rate above chance level (1/N where N is the number of subjects—2.3%) meaning that they all explained substantial subject‐specific variability. There was again an asymmetry effect in BA 44/45 with the left hemisphere being more subject‐specific than the right hemisphere for the dominant connectopy, with this effect disappearing in the second gradient. In general, mate‐based retrieval rates were higher for BA 44/45 than for V1 (despite having similar cross session ICC's), indicating that this region of the association cortex has a subject‐specific connectivity fingerprint whereas V1 has less variability in its white matter connections and follows a more standardized blueprint. Finally, the second gradient in V1 had a higher mate‐based retrieval rate than the first gradient. Given that the between session ICC relationship goes in the opposite direction, it is possible that the second dominant connectopy is merely explaining some of the variability not yet explained by g1 and thus is more subject‐specific.

### 3.3 Connectopic mapping accurately predicts the border between BA44 and BA45

**FIGURE 5:**
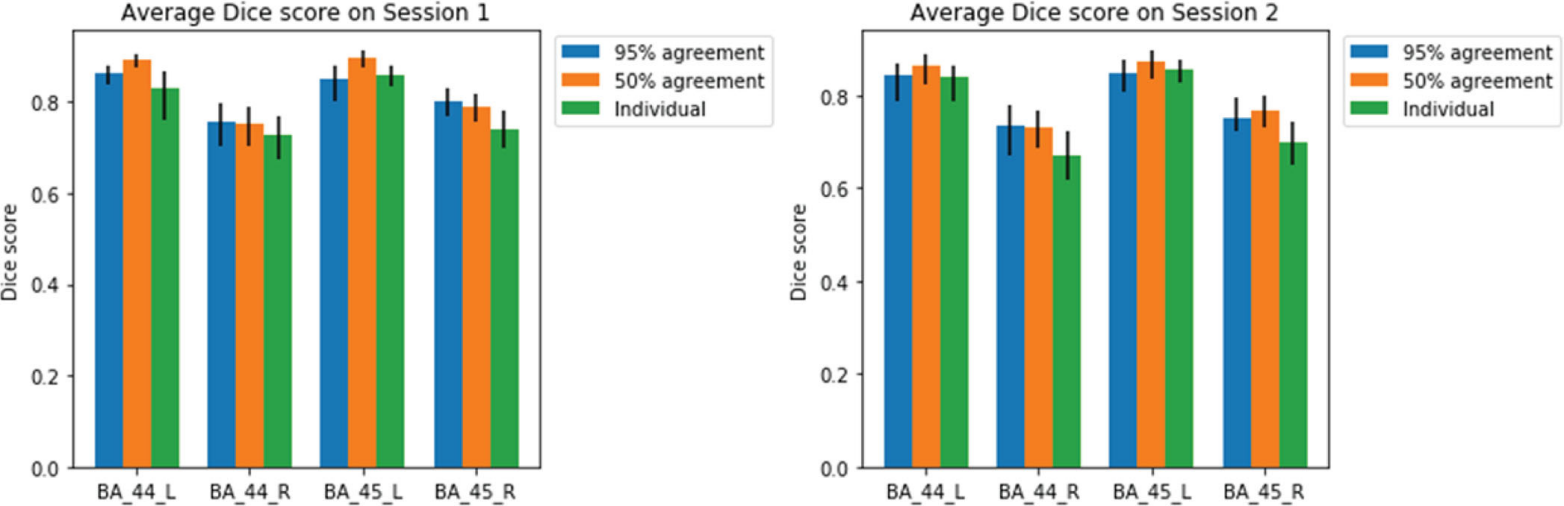
Average Dice coefficient between predicted BA44 and BA45 masks and the ground truth on the 95% agreement ROI, 50% agreement masks, and individual agreement mask in Session 1 (LEFT) and Session 2 (RIGHT). Error bars represent the 95% bootstrapped confidence interval with 10,000 samples. L, left hemisphere; R, right hemisphere

The ICC analysis indicated that the individual connectopic maps were indeed subjected specific. Given that BA44/45 is generally taken to consist of distinct areas based on cytoarchitecture, neurotransmitter receptors, and indeed connectivity (Amunts & Zilles, 2012), we investigated whether the principal gradient of connectivity could be used to describe the border between the posterior BA44 and the anterior BA45 as commonly described using traditional connectivity‐based clustering (Anwander et al., 2006; Klein et al., 2007; Neubert et al., 2014). The individual dominant connectopic maps of BA44/45 were segregated into two clusters using *k*‐means ++ (Arthur & Vassilvitskii, 2007) in order to ascertain if this region's dominant mode of connectivity (g1) accurately predicted each individual's BA44 and BA45 masks—obtained from the HCP Broadmann parcelation (Fischl et al., 2008). Additionally, the effect of outliers in the data was investigated by using dilated masks of the ROI in consideration. In all other experiments, a 95% agreement mask (created by assigning every vertex that belonged to the target ROI in at least 95% of the subjects was used). Two dilated masks were used (50% agreement and individual—assigning to the target ROI every vertex that was labeled in that ROI in at least one subject). The results are summarized in Figure 5.

Figure 5 shows that when a clustering algorithm is applied to the dominant connectivity mode of a mask containing distinct anatomical subunits, the individual components can be recovered with high accuracy. These results are in agreement with a previous study by Marquand et al. (2017) where the researchers mapped the connectivity modes of the striatum and highlighted that the principal mode recapitulated the anatomical boundaries between caudate, putamen, and nucleus accumbens. Additionally, DCs are on par with previously proposed parcellation methods of BA 44/45 using resting state fMRI (Jakobsen et al., 2016, 2018), and diffusion tractography (Klein et al., 2007) and are stable across sessions. There is a noticeable asymmetry in the results—the left hemisphere is consistently better parcellated than the right. Several studies have reported hemispheric differences in white matter pathways (mainly driven by the arcuate fascicle) with terminations in BA 44/45 (Catani et al., 2007; Eichert et al., 2018; Fernández‐Miranda et al., 2015; Vernooij et al., 2007) with the left hemisphere consistently containing increased streamline counts which may influence the algorithm's ability to accurately draw borders between these two anatomical regions.

Outliers in the mask (i.e., vertices from the 95% consensus mask that were not classified as BA 44 or BA 45) in a given individual were shown to have little influence in the final result. There is an overall increase in the average DC in the 50% agreement mask case, in relation to the benchmark (95% agreement mask) due to the increase of overall masks size and a slight decrease of the DC when the individual consensus mask is used. In this last case, there are enough outliers to drive down the Dice similarity in most cases (especially in the lower ends of the bootstrap confidence intervals) but still within the range found in previous studies aiming at parcellating this area of the inferior frontal gyrus.

Finally, we were interested in comparing our parcellation scheme to a manually labeled dataset, as the HCP Broadmann parcellation is also automatic. To this end, we ran our pipeline on an additional subset of 101 subjects from the HCP cohort corresponding to the subjects in present in a study by Jacobsen et al. (Jakobsen et al., 2016). In this study, the authors produced manually delineated masks for BA 44 and BA 45 on the left hemisphere based on sulcal markers.

**FIGURE 6:**
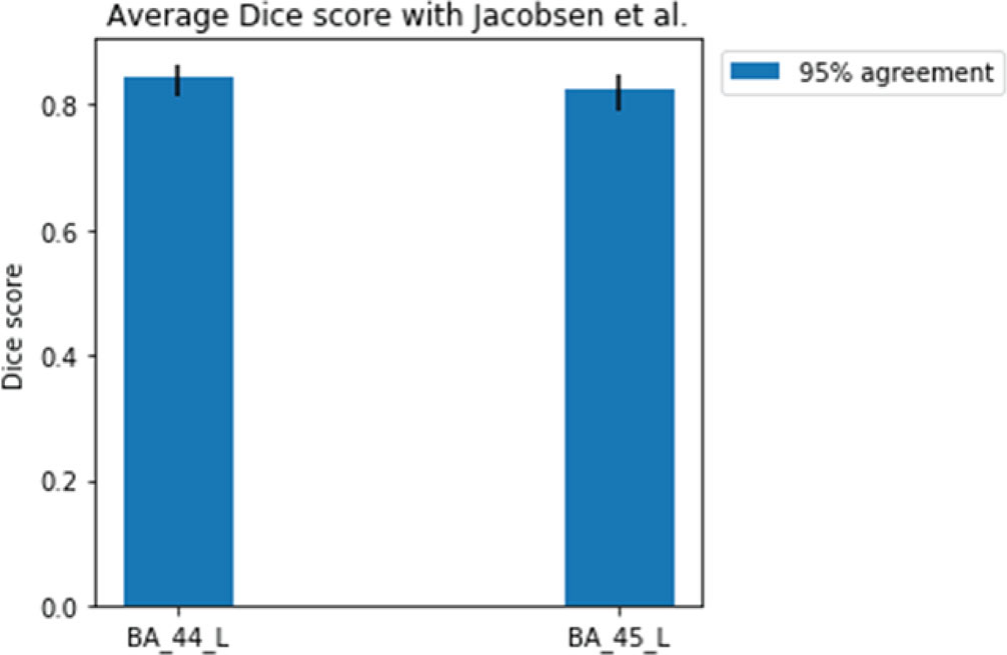
Average Dice coefficient between predicted BA44 and BA45 masks on the 95% agreement mask and the manually delineated BA 44/45 masks on the left hemisphere (Jakobsen et al., 2016) . Error bars represent the 95% bootstrapped confidence interval with 10,000 samples. L, left hemisphere

The DC of the predicted masks for BA 44/45 and the manually drawn masks in the Jakobsen et al. study (Jakobsen et al., 2016) showed (Figure 6) that this metric holds similar performance for a different cohort (different subject subset in the HCP dataset).

Taken together, these results indicate that while not its primary goal, our pipeline is suitable for finding biologically meaningful clusters derived from differences in structural connectivity between parcels.

### 3.4 Projection images reveal the origins of the observed connectivity gradients

In the previous section, we uncovered overlapping connectivity gradients in two regions of the brain, established their reproducibility and robustness, and linked them to connectivity parcellations. A key feature of the proposed pipeline is its capacity to project the individual connectivity gradient maps it reveals back onto its input data, in the form of projection images. These present a distinct advantage of using tractography data to create connectopies, as they allow one to summarize them along know tracts and thereby assess their contributions to the global connection topography of the region.

### 3.5 Projection skeletons retain known tract properties

Since these gradients can be projected back into the input space in the form of projection images, it is worth asking if the projections also maintain some of the anatomical features known of these tracts (before analyzing them further). One of the best described features of the tracts present in these projection images is their hemispheric lateralization.

In short, before projecting the gradients back onto the input space (seed × target tractography), we binarize this input space and intersect it with the tracts coming from the tract tracing software (Warrington et al., 2020). We deem a tract to be relevant toward the connectivity gradient of a given ROI if that tract intersects the tractography input space by more than 5% of its (the input space) size.

**FIGURE 7:**
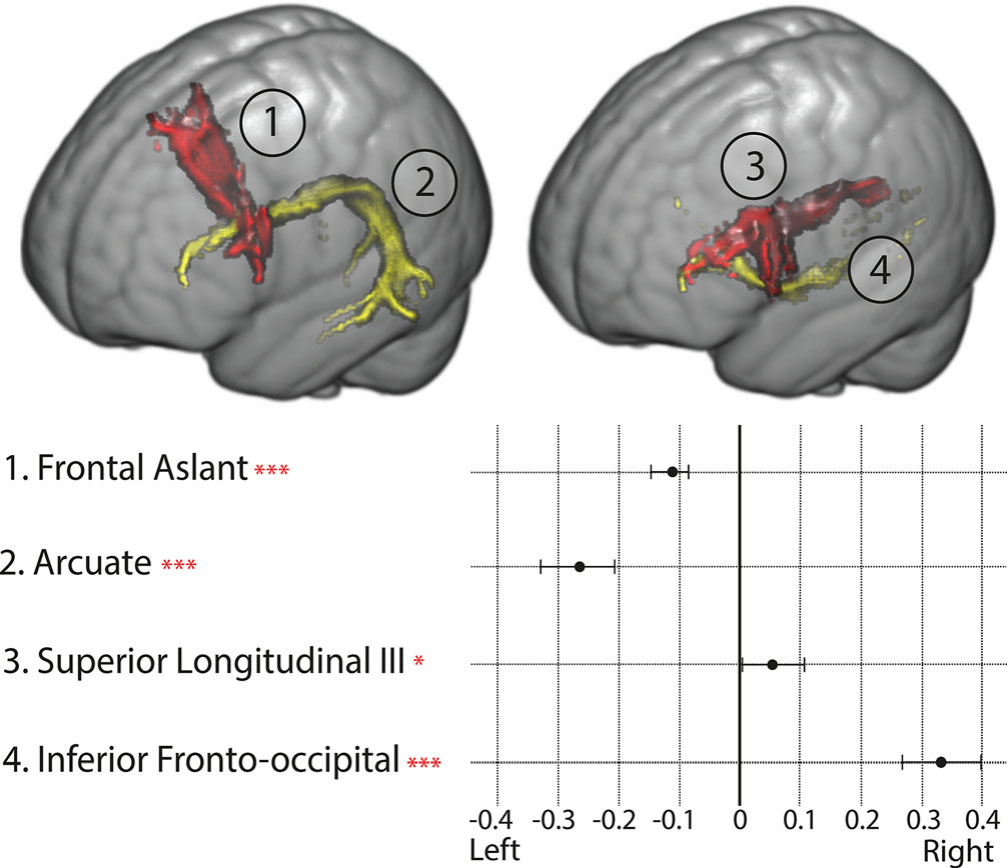
(Top) One example of each of the analyzed tracts. 1—Frontal aslant, 2—Arcuate fascicle, 3—Superior longitudinal fascicle III, 4—Inferior fronto‐occipital fascicle. (Bottom) Laterality index of relevant tracts for BA44/45's projection images. Value represents the average laterality across all subjects with brackets representing the 95% confidence interval of the mean. *** *p* < .0005, *p < .05 after Bonferroni correction (one sample *t*‐test)

We found that the gradients in BA44/45 were driven by the third branch of the superior longitudinal fascicle (SLF3), arcuate fascicle, frontal aslant, and Inferior fronto‐occipital fascicle (IFOF). The lateralization index of these tracts was defined by a ratio between the tract volume in the left and right hemisphere (Thiebaut de Schotten et al., 2011). The lateralization index of the tracts involved in BA44/45 connectivity signature can be found in Figure 7, which shows that the frontal aslant and the arcuate fascicle are left lateralized, whereas the SLF3 and the IFOF are right lateralized. These results reflect those of previous studies on the frontal aslant (Catani et al., 2012), arcuate fascicle (Catani et al., 2007), SLF3 (Howells et al., 2018), and IFOF (Hau et al., 2016).

**FIGURE 8:**
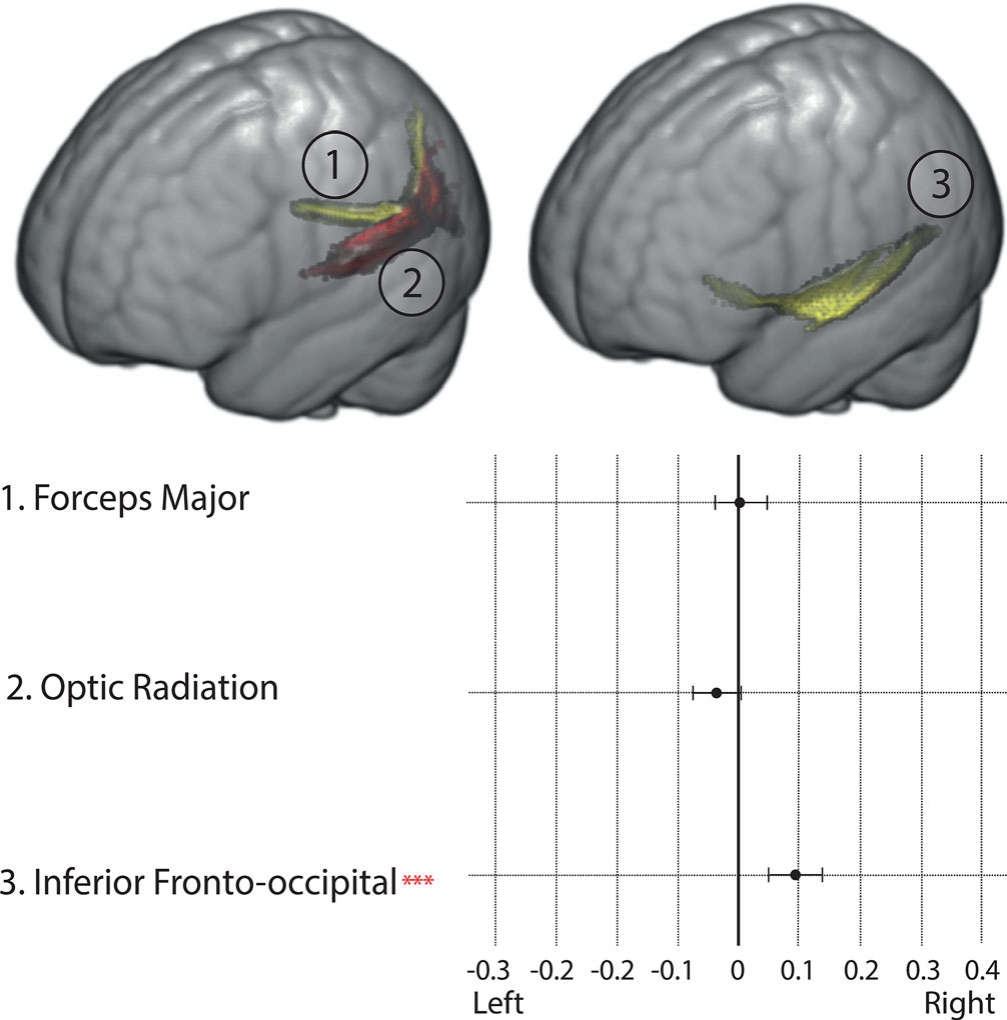
(Top) Illustration or one example of each of the analyzed tracts. 1—Forceps major, 2—Optic radiation, 3—Inferior fronto‐occipital fascicle. (Bottom) Laterality index of relevant tracts for V1's projection images. Value represents the average laterality across all subjects with brackets representing the 95% confidence interval of the mean. ***p < .0005, *p < .05 after Bonferroni correction (one sample *t*‐test)

We found that the gradients in V1 were driven by the optic radiation, forceps major, and IFOF. Figure 8 shows that there is no significant lateralization of the forceps major or the optic radiation (the optic radiation was significantly left lateralized in the retest cohort—Supporting Information) and that the IFOF is right lateralized. These results corroborate previous studies on the forceps major (Johnson et al., 2014), optic radiation (Bürgel, Schormann, Schleicher, & Zilles, 1999), and IFOF (Hau et al., 2016).

The results presented in Figures 7 and 8 indicate that the input to the proposed connectopic mapping pipeline preserves previously reported anatomical properties of the underlying white matter connectome.

### 3.6 Projection images and tract projections separate the contributions of each tract to the connectivity signature

**FIGURE 9:**
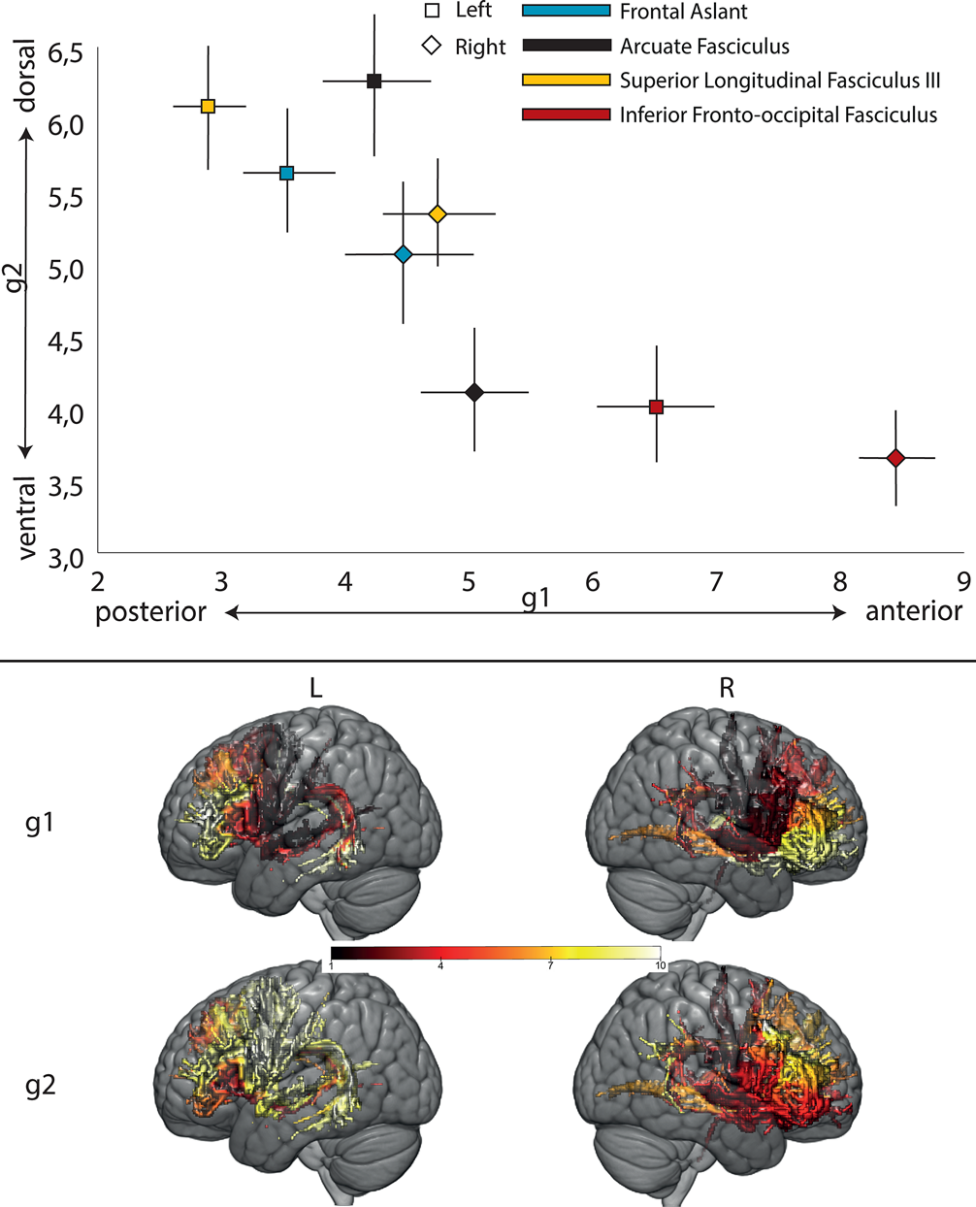
(Top) Average value of BA 44/45's examined white matter tract projections. Error bars represent the bootstrapped 95% confidence interval of the mean. *X* axis—Value along the projection image of the dominant connectivity mode (g1). For clarity of interpretation, the direction of the corresponding gradient is indicated under the axis; *Y* axis—Value along the projection image the second dominant connectivity mode (g2). For clarity of interpretation, the direction of the corresponding gradient is indicated to the left of the axis. (Bottom) Projection images for a representative subject. L and R denote left and right hemispheres, respectively

**FIGURE 10:**
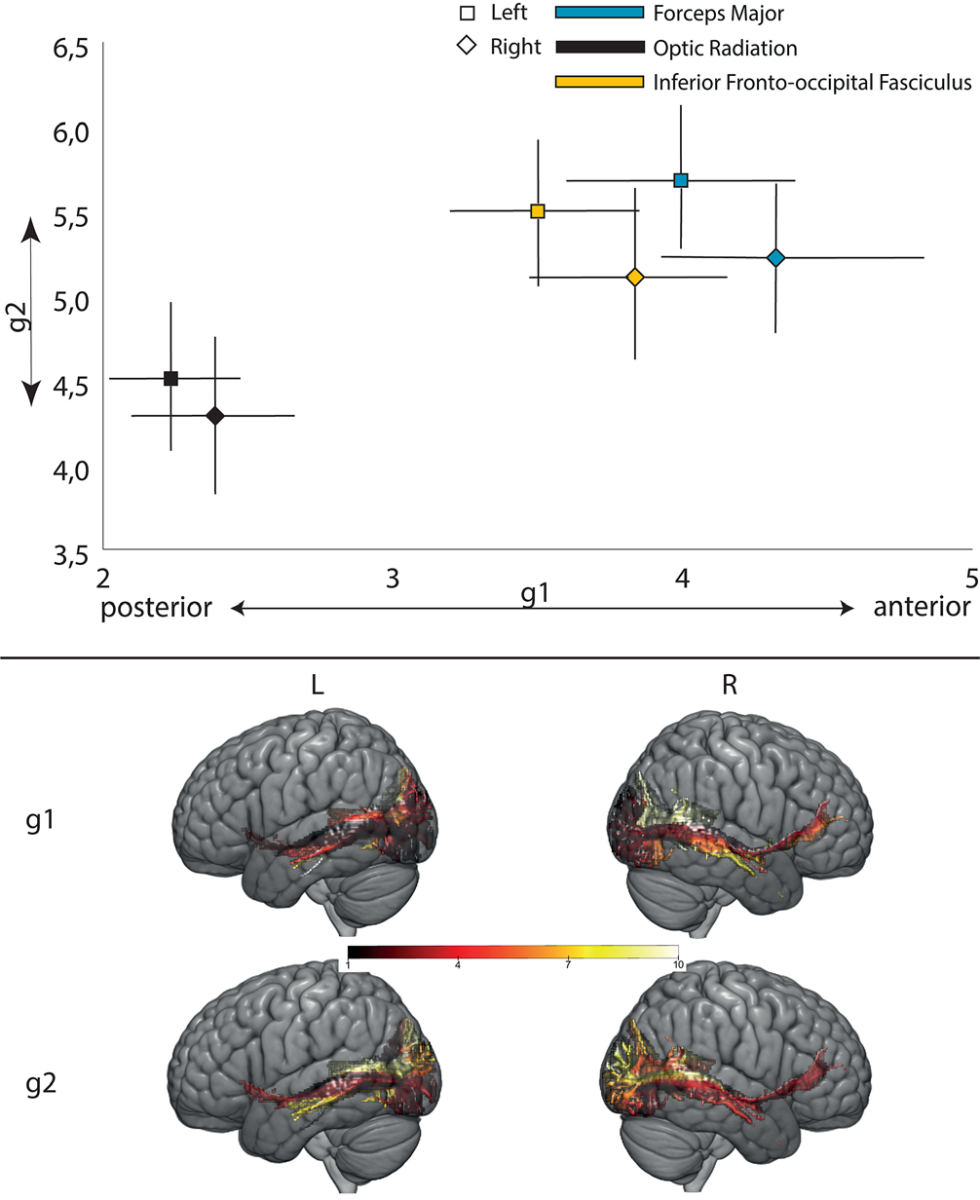
(Top) Average value of V1's examined white matter tract projections. Error bars represent the bootstrapped 95% confidence interval of the mean. *X* axis—Value along the projection image of the dominant connectivity mode (g1). For clarity of interpretation, the direction of the corresponding gradient is indicated under the axis; *Y* axis—Value along the projection image the second dominant connectivity mode (g2). For clarity of interpretation, the direction of the corresponding gradient is indicated to the left of the axis. (Bottom) Projection images for a representative subject. L and R denote left and right hemispheres, respectively

In the previous section, we evaluated if the tracts found in projection images retained known anatomical features. Here, Figures 9 and 10 show the distribution of values in each tract projection within the projection images resulting from the two dominant connectivity modes of BA 44/45 and V1 respectively. It should be noted the all connectopic maps are normalized between 1 and 10 (the original scale is between −3E‐3 to 3E‐3—normalization was necessary for plotting and alignment between subjects), which then is reflected in the scaling of the projection images.

Figure 9 highlights the contributions of each tract to the underlying connectome of BA 44/45. There is asymmetry in the tract projections themselves (the same tract in different hemisphere does not project in similar positions in this projection space) and where they are separable (the left hemisphere tracts are spread along the first dimension whereas the right hemisphere tracts lay across the second dimension). This is in agreement with the results presented in Figure 7, highlighting the lateralization of all tracts in the connectome of BA 44/45 and presents more evidence to the already extensively studied hemispherical differences in anatomy (Uylings, Jacobsen, Zilles, & Amunts, 2006), connectivity (Tomasi & Volkow, 2012) and function (Binder et al., 1997) of BA 44/45.

The posterior end of the dominant gradient (*x* axis) most strongly maps on to the frontal aslant, the arcuate fascicle, and SLF3 projections. In the left hemisphere, these posterior tract projections are separable in the gradient (meaning that there is minimal overlap between the 95% confidence interval of the tract projection means in this axis) and the arcuate occupies a position anterior to both the SLF3 and the frontal aslant. This is congruent with the notion that the SLF3 (Friederici, 2009) and the frontal aslant (Catani et al., 2012) have terminations in BA44 (posterior end of BA 44/45) and that the Arcuate Fascicle innervates both BA 44 and BA 45 (Anwander et al., 2006; Eichert et al., 2018). Still in the left hemisphere, the anterior end of the dominant gradient is occupied by the Inferior fronto occipital fascicle projection, confirming earlier work that located IFOF terminations in pars triangularis (Anwander et al., 2006). In the right hemisphere, this disposition is repeated with the exception that the more anterior tracts (arcuate fascicle, IFOF, and SLF) are no longer separable; suggesting a larger intersection of the terminations of these tracts in BA 44 on the right hemisphere consistent with the lower DCs of BA 44 compared to BA 45 on the right hemisphere dominant BA44/45 gradient (Tables 3 and 4).

**TABLE 3:** Reproducibility of connectopic mapping of the OR at the single‐subject level

ICC	OR (g1)	OR (g2)
Between sessions	L—0.696 [0.599–0.770]	L—0.294 [0.159–0.415]
	R—0.569 [0.424–0.667]	R—0.161 [0.074–0.307]
Between subjects		
Session 1	L—0.704 [0.687–0.720]	L—0.287 [0.260–0.315]
	R—0.612 [0.594–0.628]	R—0.200 [0.173–0.226}
Session 2	L—0.650 [0.630–0.670]	L—0.159 [0.131–0.186]
	R—0.379 [0.349–0.409]	R—0.071 [0.041–0.099]

*Note*: Results are compared between sessions from the same subject in both sessions and between pairs of subjects within a session. Reported values represent the average intra‐class correlation coefficient across same subject pairs in different sessions (between sessions) or different subject pairs in the same session (between subjects). Values between square brackets indicate the lower and upper bounds of the bootstrapped 95% confidence interval with 10,000 samples, respectively.

Abbreviations: L, left hemisphere gradient; R, right hemisphere gradient.

**TABLE 4:** Mate‐Based retrieval rate for first and second dominant connectopies for the OR

Mate‐based retrieval rate	OR (g1)	OR (g2)
Between sessions	L—6.8% (13.6%)	L—4.5% (11.3%)
	R—4.5% (11.4%)	R—13.6% (20.5%)

*Note*: Reported values represent the percentage of subjects, for which the connectopy in one session was maximally correlated to the corresponding connectopy in the other session. Values in brackets represents the same measure, but allowing for the correspondent connectopy to be in the top three matches.

Abbreviations: L, left hemisphere; R, right hemisphere.

The second dominant gradient (*y* axis in Figure 9) also separates the frontal aslant, arcuate fascicle, and SLF3 projections from the IFOF. However, inspection of the second dominant connectopic map reveals that this dimension is separating tract projections in terms of their placement on a ventral to dorsal axis. One key hemispheric difference of the tract projections in the second dominant gradient is visible on the arcuate fascicle. In the left hemisphere, this tract projection lies more dorsally and is separable from the IFOF tract projection whereas in the right hemisphere, this difference is attenuated by a more ventral disposition of the arcuate regarding its projection.

Overall, these results taken together with the BA 44/45 group connectopic maps in Figure 3 suggest that BA 44/45 contains at least two overlapping modes of structural connectivity change resulting from its topographically organized white matter connections.

The dominant mode of connectivity is a posterior–anterior gradient with a sharp connectivity change around the border of these two Brodmann areas. BA 44 includes the frontal aslant, and SLF while the arcuate fascicle innervates both areas 44 and 45 with the IFOF projecting to mainly area 45. Functionally, this gradient of connectivity overlaps with a task gradient in the same space where semantic unification recruits BA45, syntactic unification is spread through BA45 and BA44 and phonological processes activate BA44 (Hagoort, 2013).

The second dominant mode of connectivity represents a ventral‐dorsal gradient of connectivity separating ventral from dorsal tracts. This disposition is consistent with theories proposing a dual pathway model (ventral and dorsal) for language processing in the brain linking BA 44/45 to temporal lobe language areas. Dorsally, the SLF3 and arcuate fascicle have an important role in speech repetition and complex syntactic processes respectively and more ventrally, the IFOF has a key role in semantic processing (Friederici et al., 2013).

Figure 10 paints a different picture for the tract projections resulting from the connectopic maps in primary visual cortex. Compared to BA 44/45, these tract projections present greater symmetry, meaning that the corresponding tract projections from different hemispheres cluster together. This means that the contributions of white matter tracts to the primary visual cortex connectome are similar in both hemispheres, a key feature of the symmetric vision function in the brain (Haak et al., 2017; Rokem et al., 2017; Wu & Wu, 2017) which was also shown in Figure 8, with only the IFOF presenting significant volume lateralization.

The dominant connectivity mode places the tract projections of the optic radiation in a more posterior part of V1 with the tract projections from the IFOF and the Forceps Major lying in a more anterior position. There is a striking correspondence between the projection of these tracts along the dominant connectivity mode of V1 and its role in eccentricity mapping (Haak et al., 2017). The optic radiation, which receives input from the contralateral optic nerve, projects preferentially to the posterior end of V1, in the foveal end of the retinotopic eccentricity map (Daniel & Whitteridge, 1961; Duncan & Boynton, 2003) while the opposite extreme, the more anterior regions of V1 and, correspondingly more peripheral positions in the eccentricity map, is projected onto by the Forceps Major which connects the two visual fields (Saenz & Fine, 2010). These results are in agreement with earlier studies of white matter connectivity in the occipital cortex and particularly, in V1 (Rokem et al., 2017; Takemura et al., 2017).

As indicated above, the second gradient did not explain significant additional variance and this is also reflected in the results from the ICC analysis and the mate‐based retrieval test. It is also reflected in its projection image. As can be seen in the y‐axis in Figure 10, it does not contribute for further separation of white matter tract contributions; collapsing these tract projections onto the *y* axis would make them indistinguishable from each other.

### 3.7 Connectopic mapping on the optic radiation cross section reveals its own topographic disposition

Thus far, we have applied connectopic mapping to connectivity of specific parts of the gray matter. However, the major white matter fibers of the brain itself have some topographical organization. The clearest case of this is the organization of the corpus callosum which is topographically organized in the anterior posterior axis, connecting specific parts of the cortex with their contralateral homolog (Genç, 2011), but overlapping gradients of connectivity have also been observed in coronal cross sections of the optic radiation (Aydogan & Shi, 2016; Aydogan & Shi, 2018; Kammen, Law, Tjan, Toga, & Shi, 2016). The optic radiation plays a key role in the retinofugal pathway that connects the retina to the primary visual cortex (V1) through the lateral geniculate nucleus (LGN). The LGN is connected to V1 via the optic radiation and, as previous work has shown, its terminations in V1 are topographically organized (Aydogan & Shi, 2018), providing an anatomical basis for the eccentricity and polar angle modes of this primary cortex region. We therefore investigated the method's capability of identifying these connection topographies and how they projected onto the full tract. In essence, after tracking the optic radiation for each subject, we performed connectopic mapping on a cross‐section of this tract along the *y* axis (rostral–caudal), using the cross‐section itself as a seed, and the remainder of the tract as the target.

**FIGURE 11:**
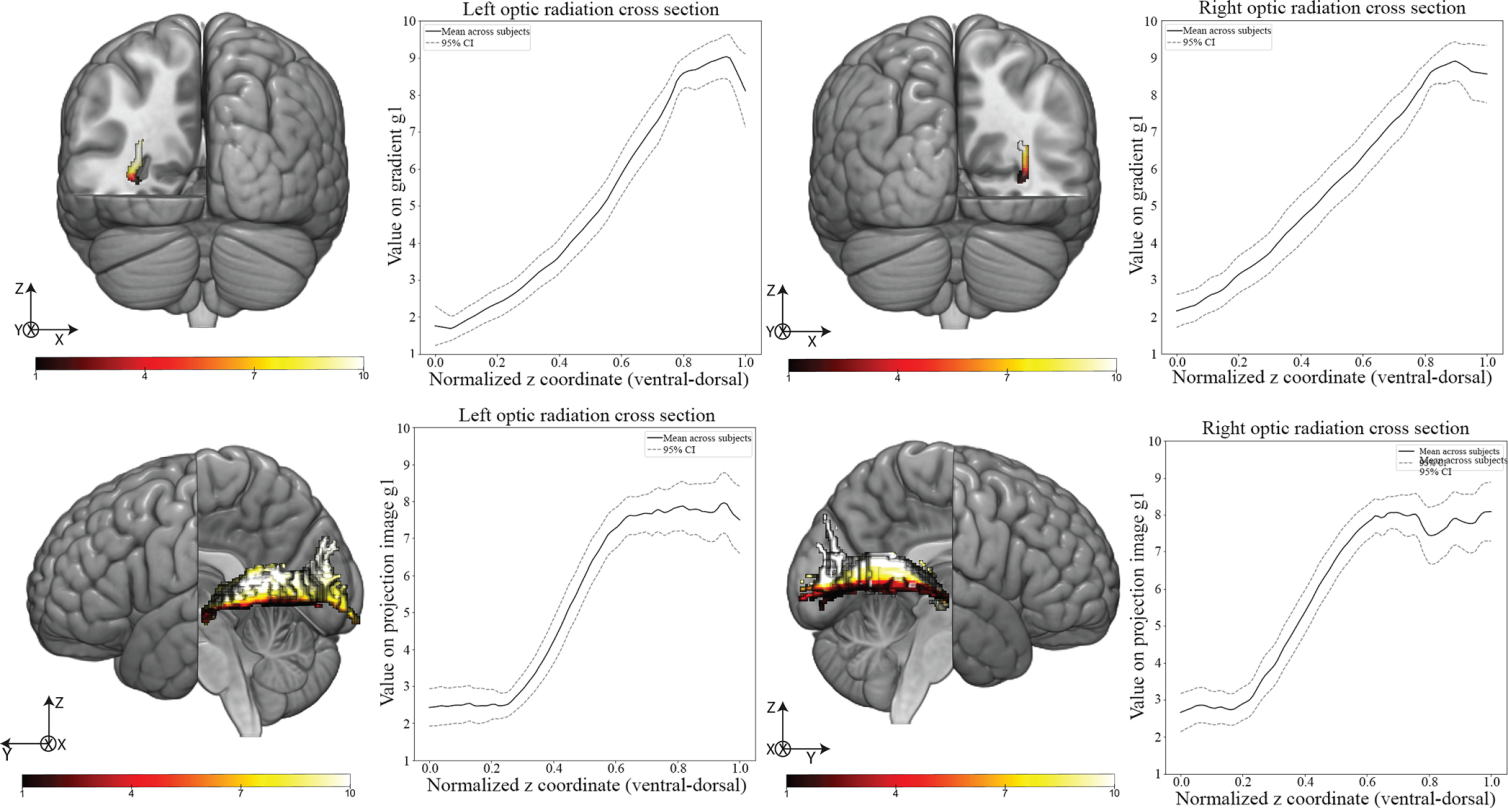
Top—(Left) Representative subjects' left seed gradient cross section (Graph) mean value of gradient g1 (dominant connectivity mode) along the z‐axis on the left optic radiation cross section. (Right) Representative subjects' right seed gradient cross section (Graph) mean value of gradient g1 (dominant connectivity mode) along the z‐axis on the right optic radiation cross section. Gradient values are normalized between 1 and 10 and the normalized z coordinate represents the range of coordinates of each subject's optic radiation cross section up sampled to 100 data points. The dashed line represents the 95% confidence interval. Bottom—(Left) Representative subjects' left projection image cross section (Graph) Mean value of projection image g1 (projected dominant connectivity mode values to the target space) along the z‐axis on the left optic radiation.(Right) Representative subjects' right projection image cross section (Graph) mean value of projection image g1 (projected dominant connectivity mode values to the target space) along the *z* axis on the right optic radiation. Projection image values are normalized between 1 and 10 and the normalized *z* coordinate represents the range of coordinates of each subject's optic radiation up sampled to 100 data points. The dashed line represents the 95% confidence interval

**FIGURE 12:**
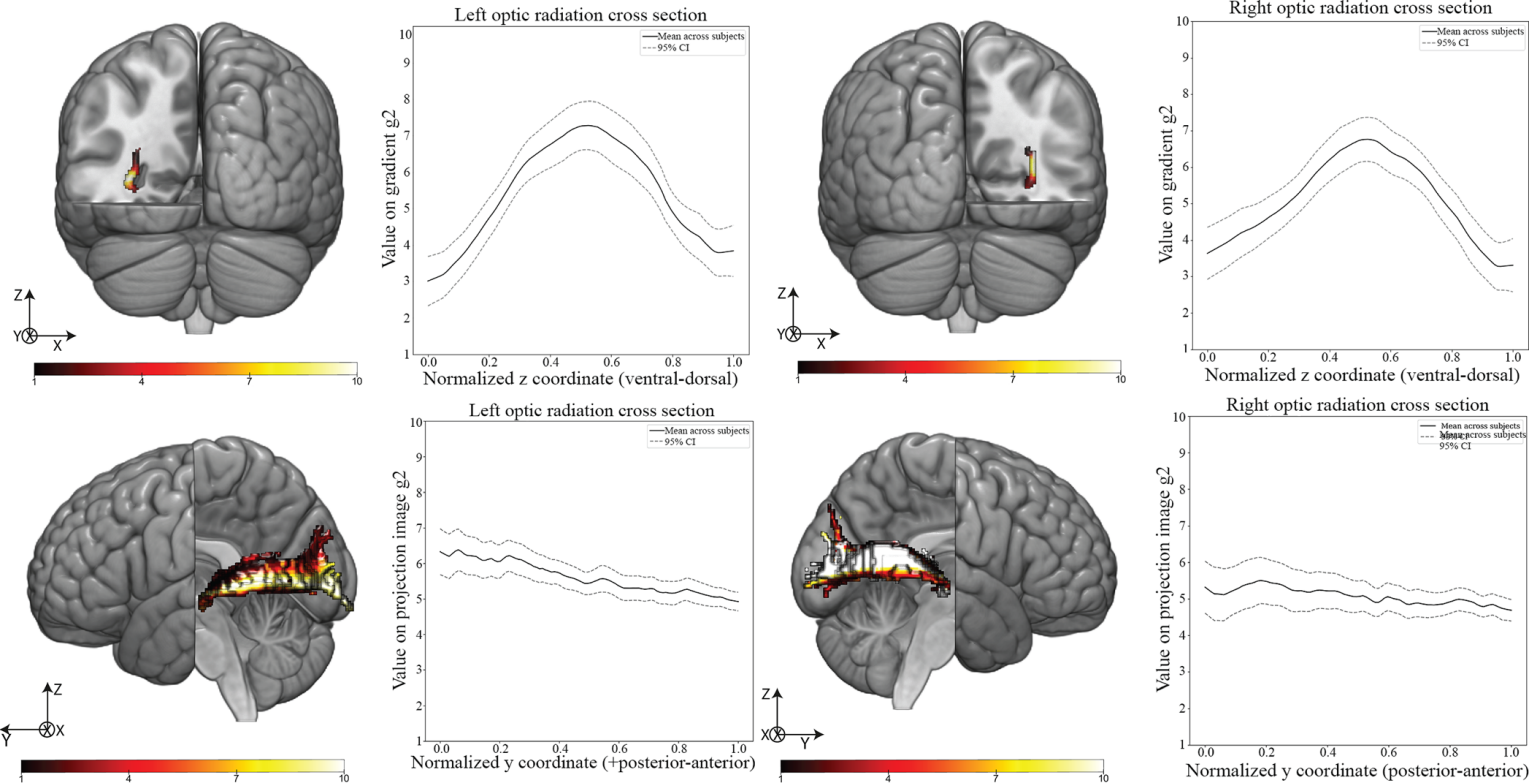
Top—(Left) Representative subjects' left seed gradient cross section (Graph) mean value of gradient g2 (second dominant connectivity mode) along the z‐axis on the left optic radiation cross section. (Right) Representative subjects' right seed gradient cross section (Graph) mean value of gradient g2 (second dominant connectivity mode) along the z‐axis on the right optic radiation cross section. Gradient values are normalized between 1 and 10 and the normalized *z* coordinate represents the range of coordinates of each subject's optic radiation cross section up sampled to 100 data points. The dashed line represents the 95% confidence interval. Bottom—(Left) Representative subjects' left projection image cross section (Graph) mean value of projection image g2 (projected second dominant connectivity mode values to the target space) along the z‐axis on the left optic radiation.(Right) Representative subjects' right projection image cross section (Graph) mean value of projection image g2 (projected second dominant connectivity mode values to the target space) along the z‐axis on the right optic radiation. Projection image values are normalized between 1 and 10 and the normalized *z* coordinate represents the range of coordinates of each subject's optic radiation up sampled to 100 data points. The dashed line represents the 95% confidence interval

The results are summarized in Figures 11 and 12 where the first two gradients of the optic radiation cross section are averaged across subjects along their normalized *z* coordinate going from ventral to dorsal. The correspondent projection images of these gradients are also shown, where the polar angle and eccentricity modes of the optic radiation are highlighted.

The dominant mode of connectivity (g1) in Figure 11 shows a bilateral linear ventral to dorsal gradient, similar to what had been observed in polar angle mode topography (Aydogan & Shi, 2018). The corresponding projection images show a ventral to dorsal gradient across the entirety of the tract, with ventral portions of it connecting to ventral areas of V1 and dorsal portions of the tract connecting to dorsal areas of V1.

The second dominant mode of connectivity (g2) in Figure 12 assumes an inverted U‐shape reinforcing the claim that the eccentricity of the fiber tracks and their coordinates on the optic radiation cross‐section should follow a U‐shape relation (Aydogan & Shi, 2016; Wärntges & Michelson, 2014). Their respective projection images confirm this topographical organization of the tract with high gradient values corresponding to fibers reaching V1 more posteriorly and low gradient values corresponding to fibers reaching V1 in a more anterior fashion.

Finally, and to evaluate the reliability of these results, an ICC analysis was made on the OR profiles, and the between‐subject ICC and between‐session ICC was computed. Additionally, a mate‐based retrieval analysis was performed, in order to access how subject specific these OR profiles were. The results are summarized in Tables 3 and 4.

Table 3 shows that there are good ICC scores between subjects and sessions for the left and right OR for gradient 1. At the session level, there is no separation between the left and right OR, which agrees with the findings highlighted in Table 1, giving more evidence for symmetric gradients in the visual system. The overall score drop for g2 is concerning and suggests there is a lot of variance within the overall trends observed for these gradients and projection images. There is also a marked difference for g2 in the right hemisphere in Session 2 either comparing it with its contralateral counterpart or with the same gradient and hemisphere in session one. This should be investigated further in a follow‐up study to determine if different noise conditions were present in this session.

Table 4 shows the same paradoxical results found in Table 2: the higher the ICC between subjects, the lower the mate‐based retrieval scores. This is caused because a higher ICC indicates a more common blueprint for that ROI, and hence, reduced identifiability. For g2, this trend is confirmed, as the mate retrieval scores increase threefold for the right hemisphere, possibly reflecting a structured noise component that is subject specific. One encouraging observation is that all values are well above chance level.

## 4 DISCUSSION

In the previous section, we demonstrated that biologically relevant, overlapping, robust, and individual connectopies can be mapped using LE of Diffusion MRI tractography data. The technique unravels overlapping modes of connectivity in a cortical ROI and tracts themselves, with its projection images highlighting each associated white matter tracts' contribution to the global connectome.

The pipeline extends previous work by Cerliani et al. (2012) and Haak et al. (2017) and was tested on 44 subjects of the Human Connectome Project. In BA 44/45 (Figure 2), two connectopies were unveiled: its dominant mode of connectivity (g1)—with a gradual change from posterior to more anterior regions and a sharper transition at the BA 44/BA 45 border area. The second dominant mode of connectivity (g2) revealed a ventral to dorsal gradient, matching the termination areas of the tracts involved in the dual pathway for language (Friederici, 2009). In V1, (Figure 3) the dominant mode of connectivity showed a posterior to anterior mapping, replicating the results obtained by applying connectopic mapping in the same region with resting state data (Haak et al., 2017) and eccentricity maps (Dougherty et al., 2003), while the second dominant mode of connectivity was shown by subsequent analysis (Table 1, Table 2 and Figure 10) to not describe any known connectivity topographies. This is a limitation of our work since at least the optic radiation (Aydogan & Shi, 2018) and the corpus callosum (Saenz & Fine, 2010) have been shown to have at least two overlapping topographically organized terminations in the primary visual cortex. This shortcoming of the pipeline—failing to identify the polar angle representation in V1 according to its global connectome can be explained by a number of factors: First, this topography exists in the tracts themselves (namely, the optic radiation) but it is masked when applying the pipeline on the global connectivity matrix of the ROI. Second, the geometry of the cortical region in question may present its own special challenges in terms of precise topographic mapping of projections: The calcarine fissure splits V1's inferior superior axis into inferior and superior portions, pushing its upper and lower bank against each other which could throw off the estimation of fiber orientation. (Rokem et al., 2017). This is exacerbated by the presence of unmyelinated (Kirilina et al., 2020) u‐fibers that can additionally impede accurate tract end points (Rokem et al., 2017). Finally, it is possible that inter‐hemispheric connections (through the Forceps major) play a major role on the polar angle (inferior–superior) mode (Saenz & Fine, 2010), but that that contribution was neglected by our decision to exclude these types of connections.

Still, these connectopic maps were shown to contain individualized, lateralized, and robust information by analysis summarized in Tables 1 and 2. Of particular interest, there is a marked leftwards lateralization of the ICC scores and mate based retrieval of the dominant connectivity mode for BA 44/45, which is in line with the notion of a common yet highly subject specific human blueprint for left‐lateralized language circuitry (Hagoort, 2014). This asymmetry effect disappears in the second dominant connectivity mode, suggesting the variability in the underlying connectome that explains the asymmetry effect is captured by the dominant connectivity mode. This is an interesting in the light of a new study that has proposed a unified ventral tract system (Weiller et al., 2021). The separation between this unified ventral tract system and the dorsal tracts projecting to BA 44/45 might be relatively symmetrical (or even rightward lateralized), causing the asymmetry effect to disappear. Projection images and their tract projections (Figures 9 and 10) are shown to reveal the separate contributions of different white matter tracts to the connectivity fingerprint and the input to the pipeline was shown to be biological coherent by performing laterality tests (Figures 4 and 5) on the white matter tracts contained in the thresholded outputs of probtrackx with all relevant white matter tracts showing lateralizations in agreement with earlier studies.

Overall, this work presents an additional solid argument for the advent of connectopic mapping. It should be noted that the results we present in Figures 4, 5, 6 are merely indicative of the fact that dimensionality reduction through LE is only a first step toward spectral clustering. We do not claim that our clustering method is more precise than any others, but rather that refraining from clustering in this connectivity reduced subspace yields relevant insights into its principles. Here and in the case of BA 44/45 for example, our pipeline shows two overlapping principles of connectivity (gradients): The first one recapitulates the anatomical borders of these two regions but the second one gives additional clues on how white matter projects there—a clear ventral‐dorsal pattern separating the dorsal and ventral pathways for language processing. In this specific case, merely clustering the connectivity matrix yields, in our view, a correct, yet incomplete view of the connectivity landscape.

The application of the pipeline onto the white matter itself using the optic radiation as case study unveiled its potential to track topographical regularities in white matter tracts. The dominant mode of connectivity (g1) and its corresponding projection images (Figure 8) were both being dominated by a linear transition system where ventral fibers on the chosen cross section projected to ventral portions of V1 and the dorsal fibers projected to dorsal V1.The second dominant of connectivity (g2) (Figure 9) showed an U‐shaped curve on the cross section of the optic radiation, as had previous studies tracking the eccentricity of the optic radiation (Aydogan & Shi, 2018). The corresponding projection images (Figure 9) confirmed this eccentricity mode of the optic radiation by corresponding higher values on g2 to posterior regions of V1 and lower values of g2 to anterior regions of V1. It is worth pointing out that there is a reversal of the order of the gradients when considering only the optic radiation (eccentricity mode appearing only on the second gradient), or V1 projections as a whole (the eccentricity mode is the main direction of connectivity switch). This seems to confirm previous findings suggesting the main direction of connectivity change in the optic radiation is indeed the dorsal‐ventral axis (Alvarez, Schwarzkopf, & Clark, 2015). The low ICC values for g2 (Table 3) warrant further investigation into this second dominant mode of connectivity for the optic radiation. The overall trends for g2 projection images should be accessed against their V1 terminations to be able to determine with more certainty their correspondence with the eccentricity mode of the primary visual cortex.

The possibility of mapping white matter dependent cortical connectopies provides a cortical window into white matter organization. When parameterized using trend surface modeling (Haak et al., 2017), these maps may provide a biomarker for disease or behavior (Marquand et al., 2017). Potentially, overlapping modes of connectivity would be differently affected by certain conditions and these changes would map onto symptom presence and severity. Changes of structural connectivity modes across lifetime would also be able to model in developing (Catani & Bambini, 2014) and aging populations. Another prospect derives from the flexibility of the pipeline: connectopies can be mapped not only from a cortical ROI to the rest of the brain, but also from cortical ROI to another cortical ROI (Vijayakumar et al., 2018), from subcortical structures to the cortex (Lambert, Simon, Colman, & Barrick, 2017; Phillips et al., 2019), and from white matter itself (in this article).

One interesting feature resulting from the segregation of tracts projections in projection images is the possibility of using projection images as the input for data driven tract separation. Some techniques have been proposed toward this end with moderate success (O'Donnell & Westin, 2007), given its importance when looking at white matter organization in species where the white matter blueprint is still unknown. Additionally, individualized projection images could provide insight on the influence of white matter organization on functional lateralization of language in the brain as these phenomena have been shown to vary with age (Szaflarski, Holland, Schmithorst, & Byars, 2006).

Cross species comparison possibilities are one of the key advantages of connectopic mapping using diffusion MRI over rsfMRI since it enables the usage of post‐mortem tissue and allows for validation with histological data. In terms of evolution, one key question to answer would be how the overlay of connectopic maps changes in homologous regions across species, and the consequences of those changes to cognitive abilities (Thivierge & Marcus, 2007). Brain function modeling would also benefit from comparative connectopic mapping as the structural connectivity topography changes across species would be promising novel experimental parameters for models of functional consequences of brain evolution. (Jbabdi et al., 2013).

Yet, while our pipeline opens the way for novel cross‐species comparisons, the fact that it makes use of diffusion tractography to unveil topographic connections in the brain should be addressed. The capability of tractography algorithms to map accurately end‐to‐end connections is still under contention due to limitations such as gyral bias and kissing and fanning fibers (Assaf, Johansen‐Berg, & Thiebaut De Schotten, 2017; Jbabdi & Johansen‐Berg, 2011). Additionally, despite the advantages of using dMRI datasets described in the introduction section, an important limitation of this type of data arises in the context of gradient analysis. As dMRI data describes monosynaptic connections, gradient analysis will not uncover gradual contributions of different networks as is possible with rsfMRI (Margulies et al., 2016). Future analysis should investigate the coupling of functional and structural connectivity gradients of connectivity especially in the light of recent evidence showing that microstructural and functional gradients are increasingly untethered in higher order cortices (Paquola et al., 2019).

While it is important to disentangle the overlapping connectivity patterns that drive connectopies, it is essential to assign a biological meaning to them(Haak & Beckmann, 2020). In the case of this pipeline, the resulting connectopic maps are back‐projected onto the input skeleton in a similar fashion employed by Haak et al. (2017). Since the input is diffusion tractography data, the ensuing projection images represent an approximation of the manifold values in the input space and reliably separate tracts in the input space. While this was evidenced in the tract projections, there is still a notable reduction of the amplitude of values in projection images given that they are formed by averaging gradient values. One possible consequence of this limitation of the current model is highlighted on the projection images for the second dominant connectopy of the optic radiation, where the already low amplitude of gradient values were even more restricted on the projection images. Future developments of the pipeline will focus in further refining the back‐projection step, with some studies already tackling this issue (Friedrich, Forkel, & de Schotten, 2020).

## Supporting information


**FIGURE S1** Group connectopic maps of BA 44/45 overlaid on an inflated cortical surface (retest cohort). The top row shows the connectopic maps for the dominant connectivity mode (g1). The bottom row shows the connectopic for the second dominant connectivity mode (g2). The L R labels refer to the left and right hemisphere respectively.

**FIGURE S2** Group connectopic maps of V1 (occipital pole plane‐ dashed line) overlaid on an inflated cortical surface (retest cohort). The top row shows the connectopic maps for the dominant connectivity mode (g1). The bottom row shows the connectopic for the second dominant connectivity mode (g2)—deemed unreliable by the dimensionality estimation algorithm. The L R labels refer to the left and right hemisphere respectively.

**FIGURE S3** (Top) One example of each of the analyzed tracts. 1—Frontal aslant, 2—Arcuate aascicle, 3—Superior longitudinal Fascicle III, 4—Inferior fronto‐occipital fascicle. (Bottom) Laterality index of relevant tracts for BA44/45's projection images. Value represents the average laterality across all subjects with brackets representing the 95% confidence interval of the mean. ***‐ *p* < .0005, *p .05 after Bonferroni correction (one sample *t*‐test)

**Figure S4** (Top) Illustration or one example of each of the analyzed tracts. 1—Forceps Major, 2—Optic Radiation, 3—Inferior fronto‐occipital fascicle (Bottom) Laterality index of relevant tracts for V1's projection images. Value represents the average laterality across all subjects with brackets representing the 95% confidence interval of the mean. ***p < .0005, *p < .05 after Bonferroni correction (one sample *t*‐test)

**FIGURE S5** (Top) Average value of BA 44/45's examined white matter tract projections. Error bars represent the bootstrapped 95% confidence interval of the mean. *X* axis—Value along the projection image of the dominant connectivity mode (g1). For clarity of interpretation, the direction of the corresponding gradient is indicated under the axis; Y axis—Value along the projection image the second dominant connectivity mode (g2). For clarity of interpretation, the direction of the corresponding gradient is indicated to the left of the axis. (Bottom) Projection images for a representative subject. L and R denote left and right hemispheres, respectively.

**FIGURE S6** (Top) Average value of V1's examined white matter tract projections. Error bars represent the bootstrapped 95% confidence interval of the mean. *X* axis—Value along the projection image of the dominant connectivity mode (g1). For clarity of interpretation, the direction of the corresponding gradient is indicated under the axis; *Y* axis—Value along the projection image the second dominant connectivity mode (g2). For clarity of interpretation, the direction of the corresponding gradient is indicated to the left of the axis. (Bottom) Projection images for a representative subject. L and R denote left and right hemispheres, respectively.

**FIGURE S7** TOP—(Left) Representative subjects' left seed gradient cross section (Graph) mean value of gradient g1 (dominant connectivity mode) along the z‐axis on the left optic radiation cross section. (Right) Representative subjects' right seed gradient cross section (Graph) Mean value of gradient g1 (dominant connectivity mode) along the z‐axis on the right optic radiation cross section. Gradient values are normalized between 1 and 10 and the normalized *z* coordinate represents the range of coordinates of each subject's optic radiation cross section up sampled to 100 data points. The dashed line represents the 95% confidence interval. Bottom—(Left) Representative subjects' left projection image cross section (Graph) mean value of projection image g1 (projected dominant connectivity mode values to the target space) along the z‐axis on the left optic radiation.(Right) Representative subjects' right projection image cross section (Graph) Mean value of projection image g1 (projected dominant connectivity mode values to the target space) along the z‐axis on the right optic radiation. Projection image values are normalized between 1 and 10 and the normalized z coordinate represents the range of coordinates of each subject's optic radiation up sampled to 100 data points. The dashed line represents the 95% confidence interval

**FIGURE S8** TOP—(Left) Representative subjects' left seed gradient cross section (Graph) Mean value of gradient g2 (second dominant connectivity mode) along the z‐axis on the left optic radiation cross section. (Right) Representative subjects' right seed gradient cross section (Graph) mean value of gradient g2 (second dominant connectivity mode) along the *z* axis on the right optic radiation cross section. Gradient values are normalized between 1 and 10 and the normalized z coordinate represents the range of coordinates of each subject's optic radiation cross section up sampled to 100 data points. The dashed line represents the 95% confidence interval.
BOTTOM ‐ (Left) Representative subjects' left projection image cross section (Graph) Mean value of projection image g2 (projected second dominant connectivity mode values to the target space) along the z‐axis on the left optic radiation.(Right) Representative subjects' right projection image cross section (Graph) mean value of projection image g2 (projected second dominant connectivity mode values to the target space) along the *z* axis on the right optic radiation. Projection image values are normalized between 1 and 10 and the normalized *z* coordinate represents the range of coordinates of each subject's optic radiation up sampled to 100 data points. The dashed line represents the 95% confidence interval.

## Data Availability

Raw and preprocessed data are available from the Human Connectome Project (www.human connectome.org). Results will be made available upon acceptance of the article in the form or a Data Sharing Collection from the Donders Repository (www.data.donders.ru.nl). Individual level results and code to produce the figures are already published in https://github.com/gfreches/congrads-tracts and https://github.com/neuroecology/MrCat.
