# Supplementary figures and images for: Connectivity gradients on tractography data: Pipeline and example applications
Source: Hum Brain Mapp. 2021 Sep 24;42(18):5827–45. doi: 10.1002/hbm.25623 (PMC8596970; doi:10.1002/hbm.25623)

L

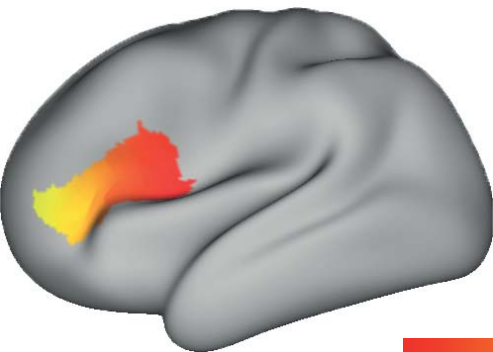

R

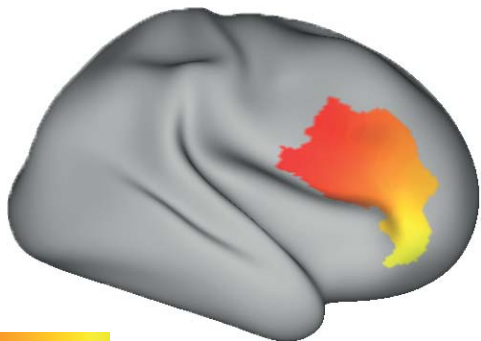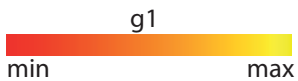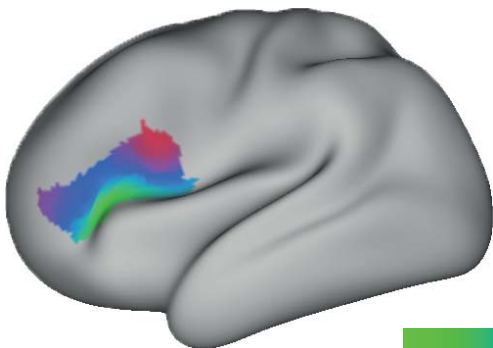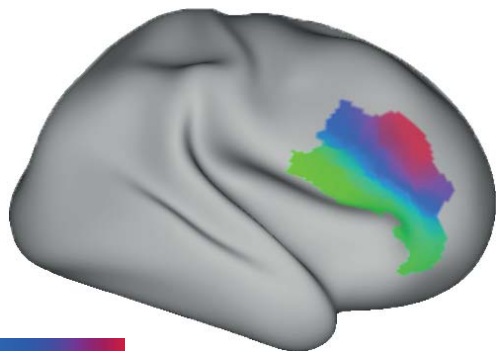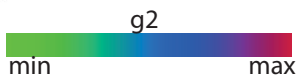

Supplement: Supplementary file 1 — FIGURE S1 Group connectopic maps of BA 44/45 overlaid on an inflated cortical surface (retest cohort). The top row shows the connectopic maps for the dominant connectivity mode (g1). The bottom row shows the connectopic for the second dominant connectivity mode (g2). The L R labels refer to the left and right hemisphere respectively. [file HBM-42-5827-s008.pdf]

L

R

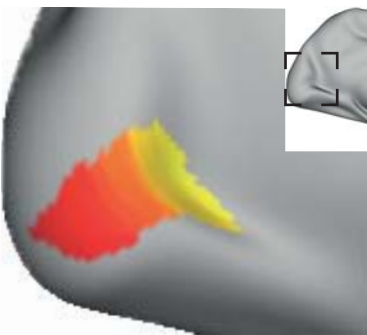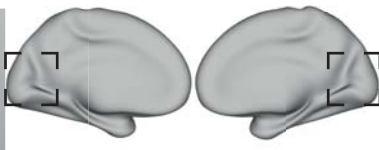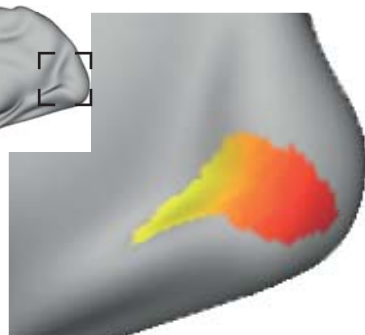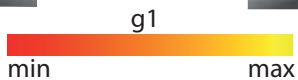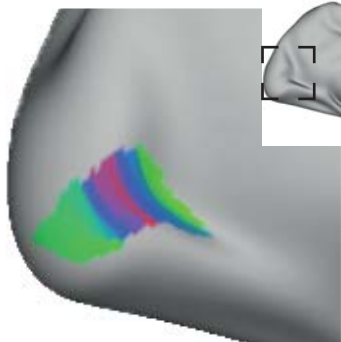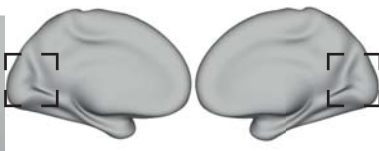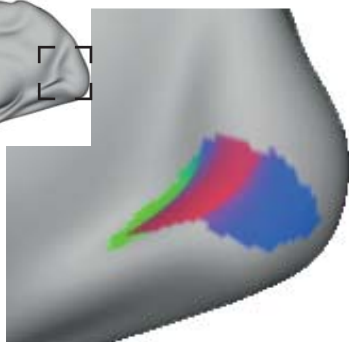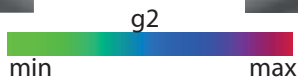

Supplement: Supplementary file 2 — FIGURE S2 Group connectopic maps of V1 (occipital pole plane‐ dashed line) overlaid on an inflated cortical surface (retest cohort). The top row shows the connectopic maps for the dominant connectivity mode (g1). The bottom row shows the connectopic for the second dominant connectivity mode (g2)—deemed unreliable by the dimensionality estimation algorithm. The L R labels refer to the left and right hemisphere respectively. [file HBM-42-5827-s004.pdf]

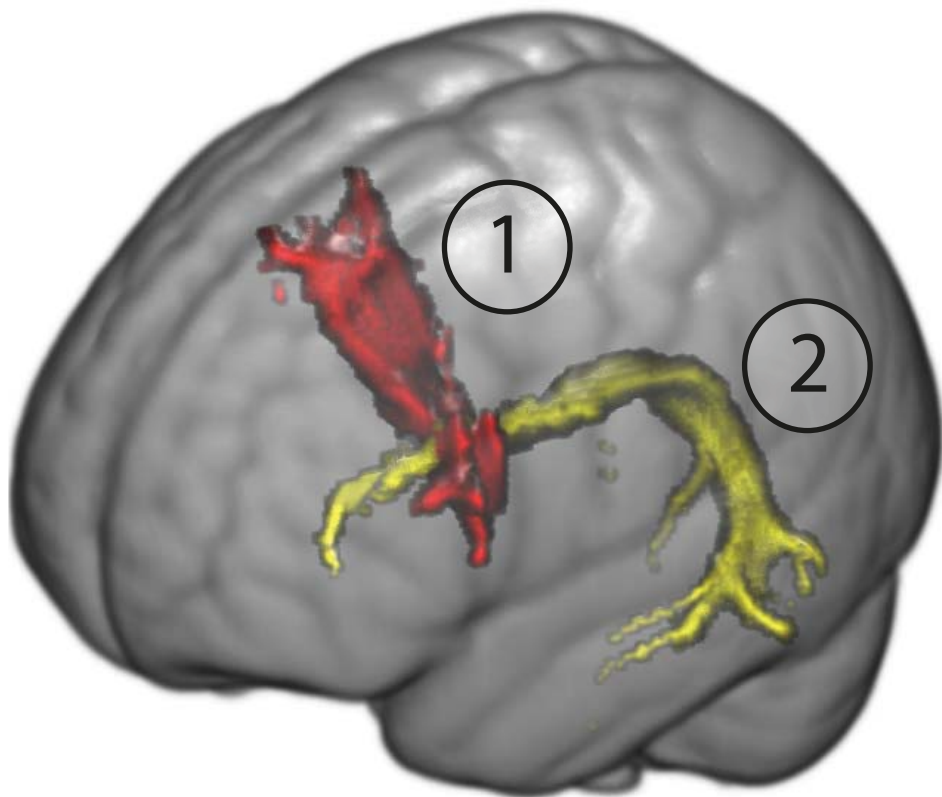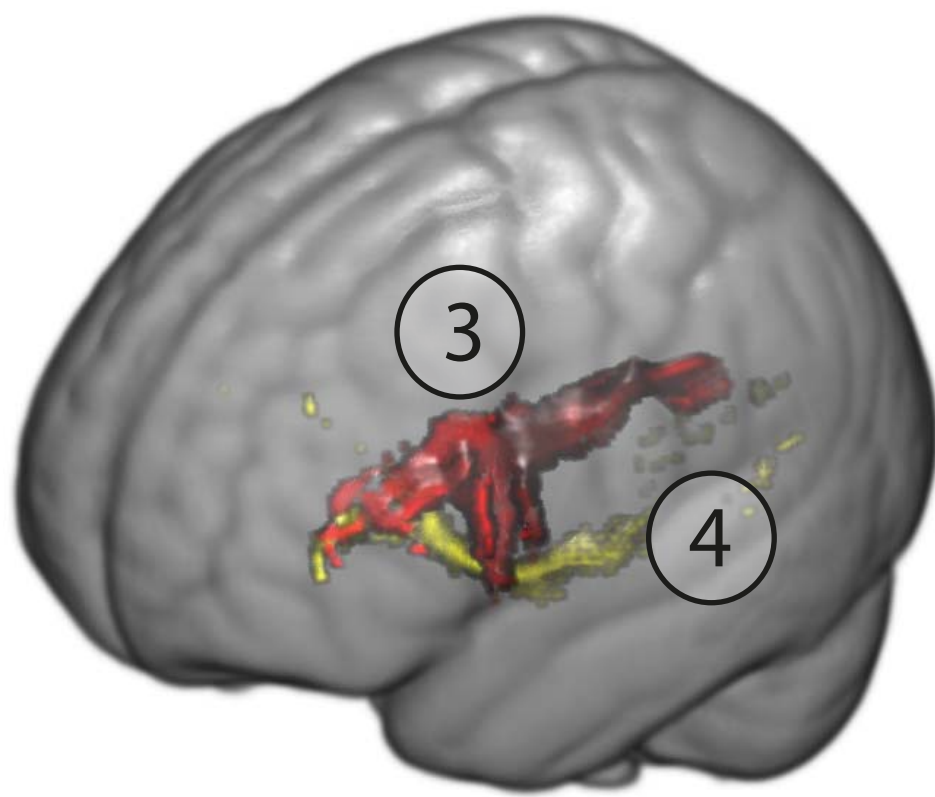

1. Frontal Aslant \*\*\*

2. Arcuate \*\*\*

3. Superior Longitudinal III \*

4. Inferior Fronto-occipital \*\*\*

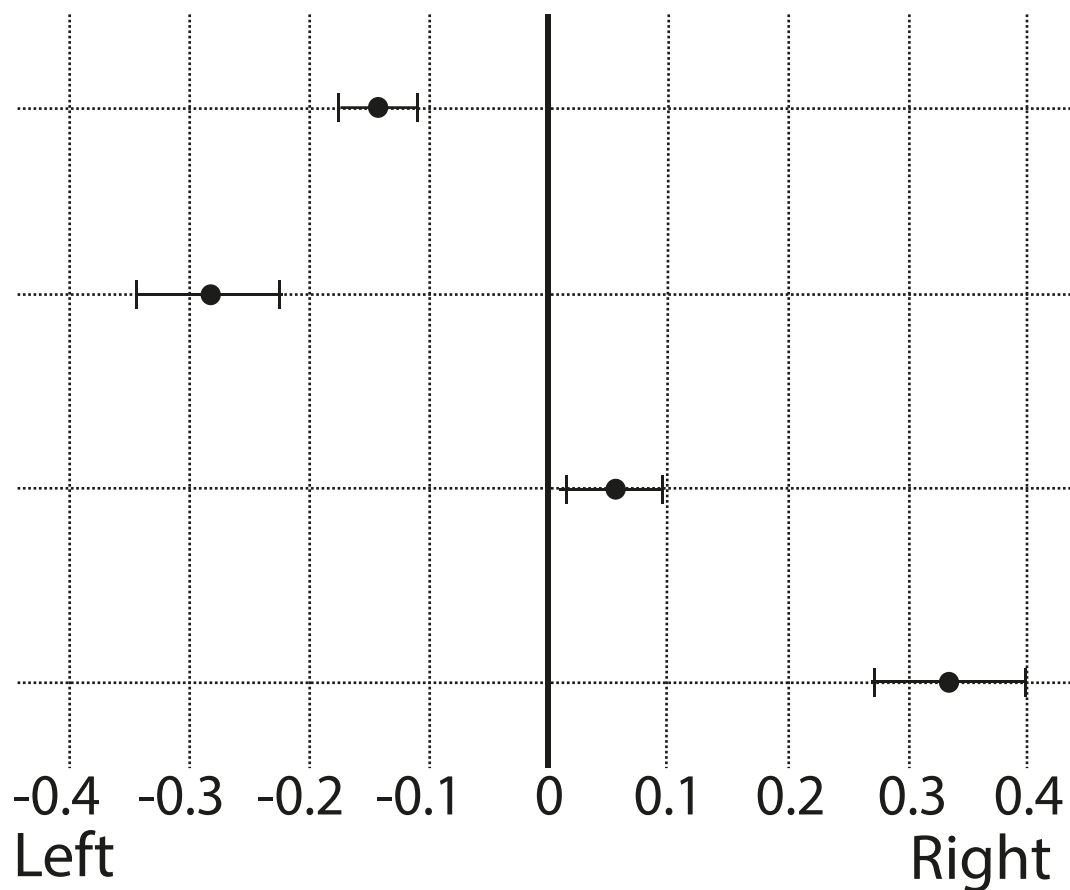

Supplement: Supplementary file 3 — FIGURE S3 (Top) One example of each of the analyzed tracts. 1—Frontal aslant, 2—Arcuate aascicle, 3—Superior longitudinal Fascicle III, 4—Inferior fronto‐occipital fascicle. (Bottom) Laterality index of relevant tracts for BA44/45's projection images. Value represents the average laterality across all subjects with brackets representing the 95% confidence interval of the mean. ***‐ p < .0005, *p .05 after Bonferroni correction (one sample t‐test) [file HBM-42-5827-s003.pdf]

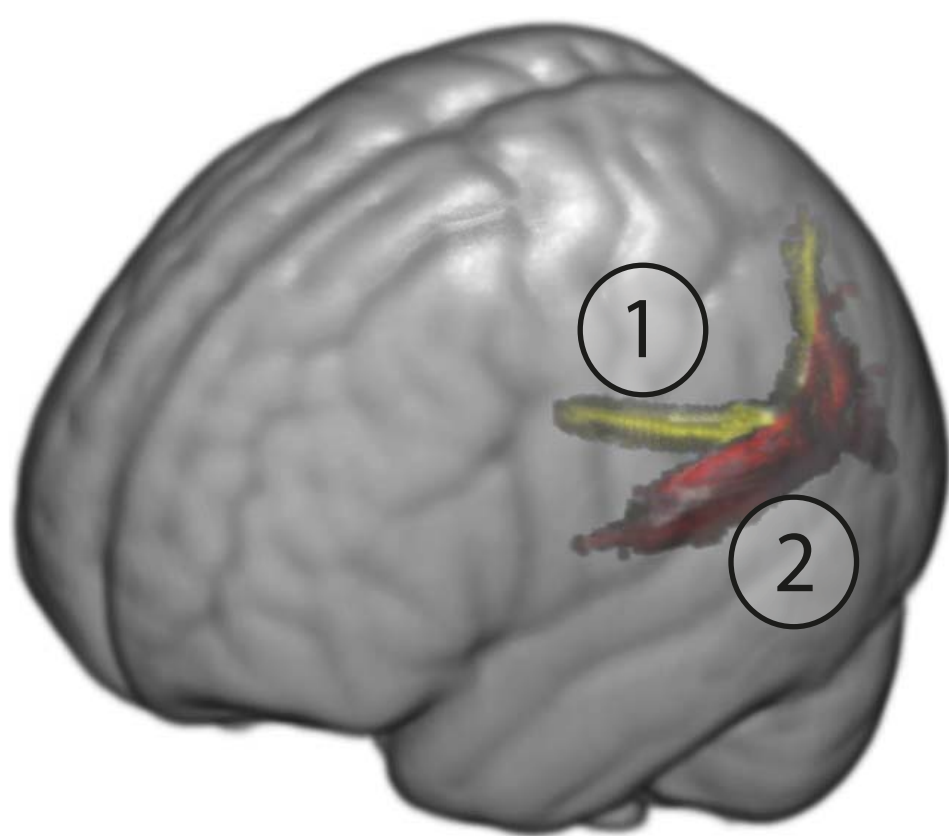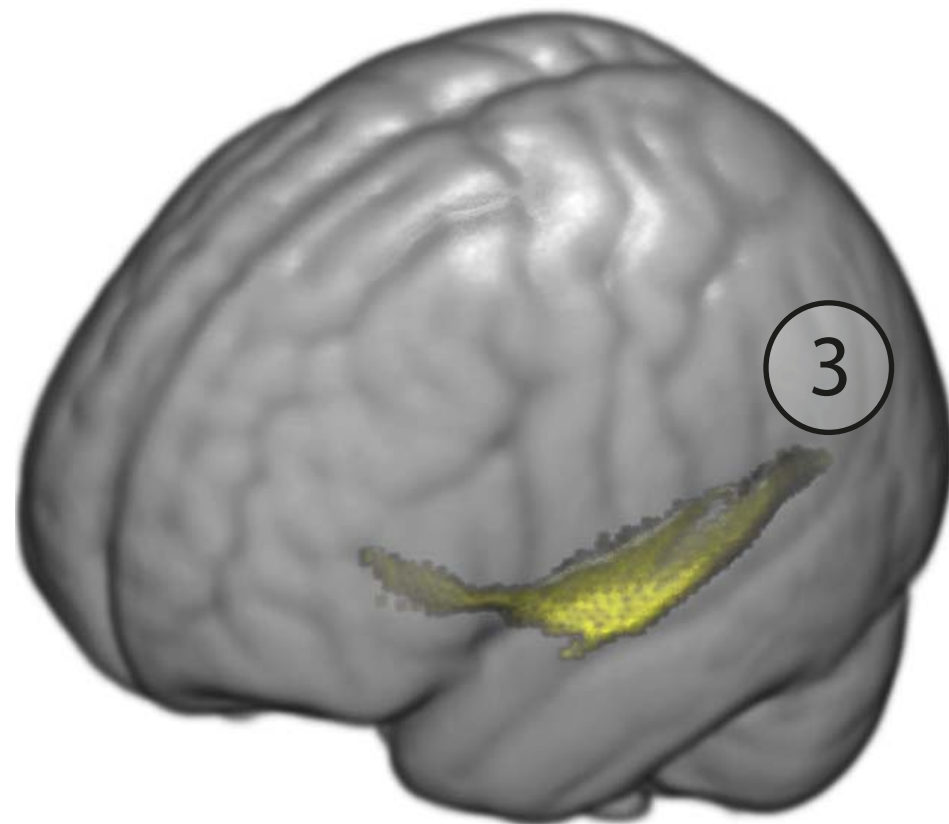

1. Forceps Major

2. Optic Radiation \*

3. Inferior Fronto-occipital \*\*\*

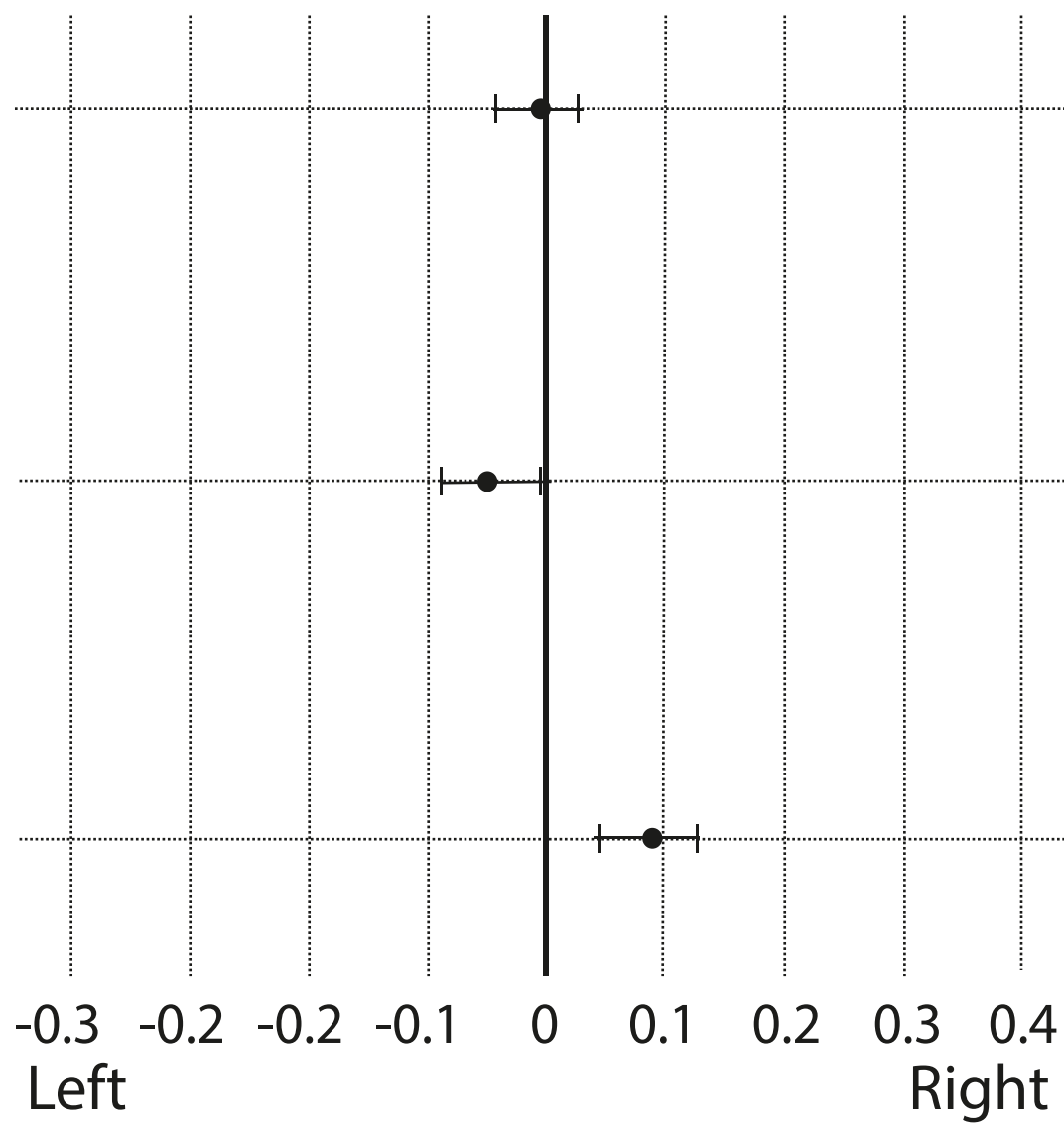

Supplement: Supplementary file 4 — Figure S4 (Top) Illustration or one example of each of the analyzed tracts. 1—Forceps Major, 2—Optic Radiation, 3—Inferior fronto‐occipital fascicle (Bottom) Laterality index of relevant tracts for V1's projection images. Value represents the average laterality across all subjects with brackets representing the 95% confidence interval of the mean. ***p < .0005, *p < .05 after Bonferroni correction (one sample t‐test) [file HBM-42-5827-s007.pdf]

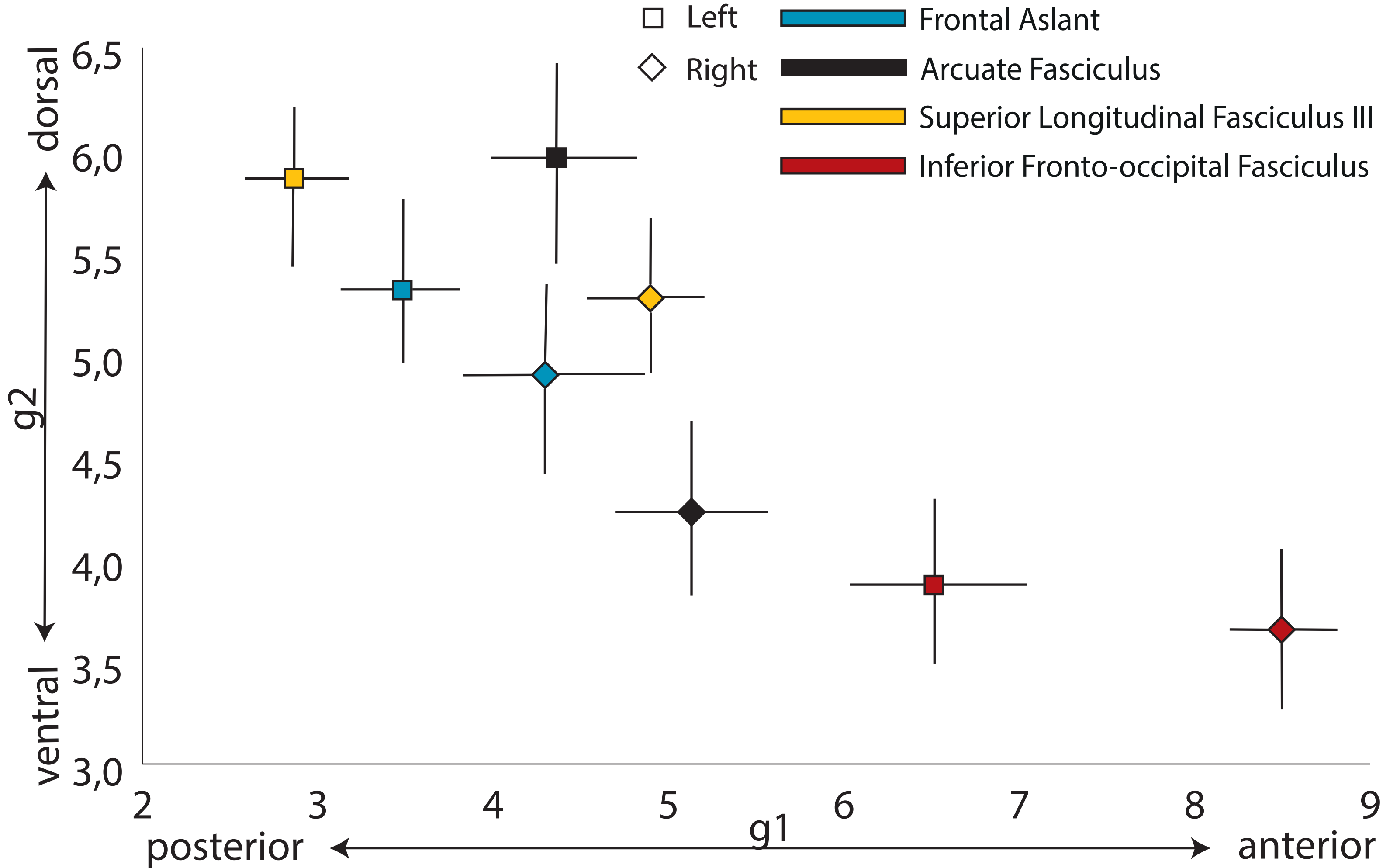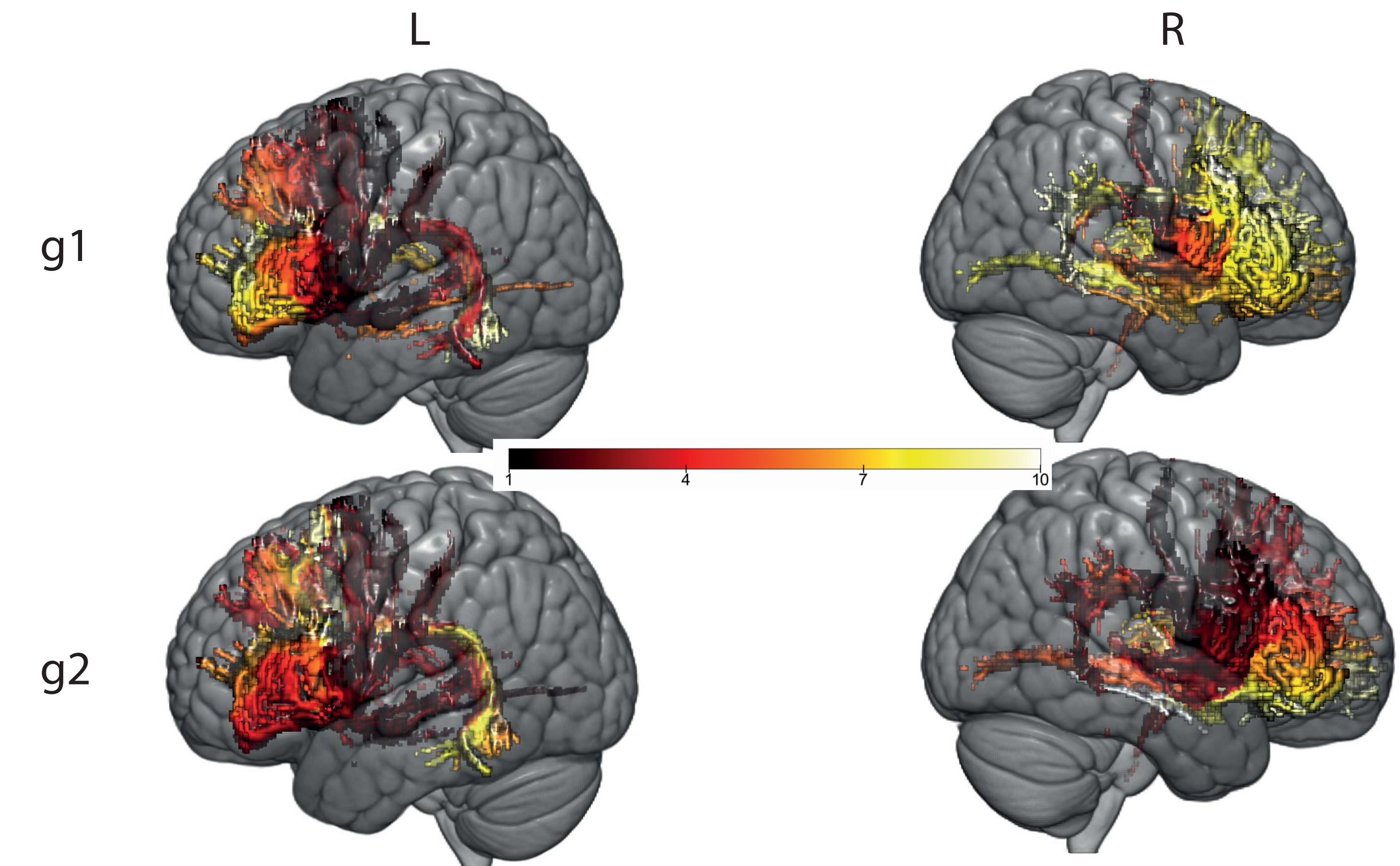

Supplement: Supplementary file 5 — FIGURE S5 (Top) Average value of BA 44/45's examined white matter tract projections. Error bars represent the bootstrapped 95% confidence interval of the mean. X axis—Value along the projection image of the dominant connectivity mode (g1). For clarity of interpretation, the direction of the corresponding gradient is indicated under the axis; Y axis—Value along the projection image the second dominant connectivity mode (g2). For clarity of interpretation, the direction of the corresponding gradient is indicated to the left of the axis. (Bottom) Projection images for a representative subject. L and R denote left and right hemispheres, respectively. [file HBM-42-5827-s006.pdf]

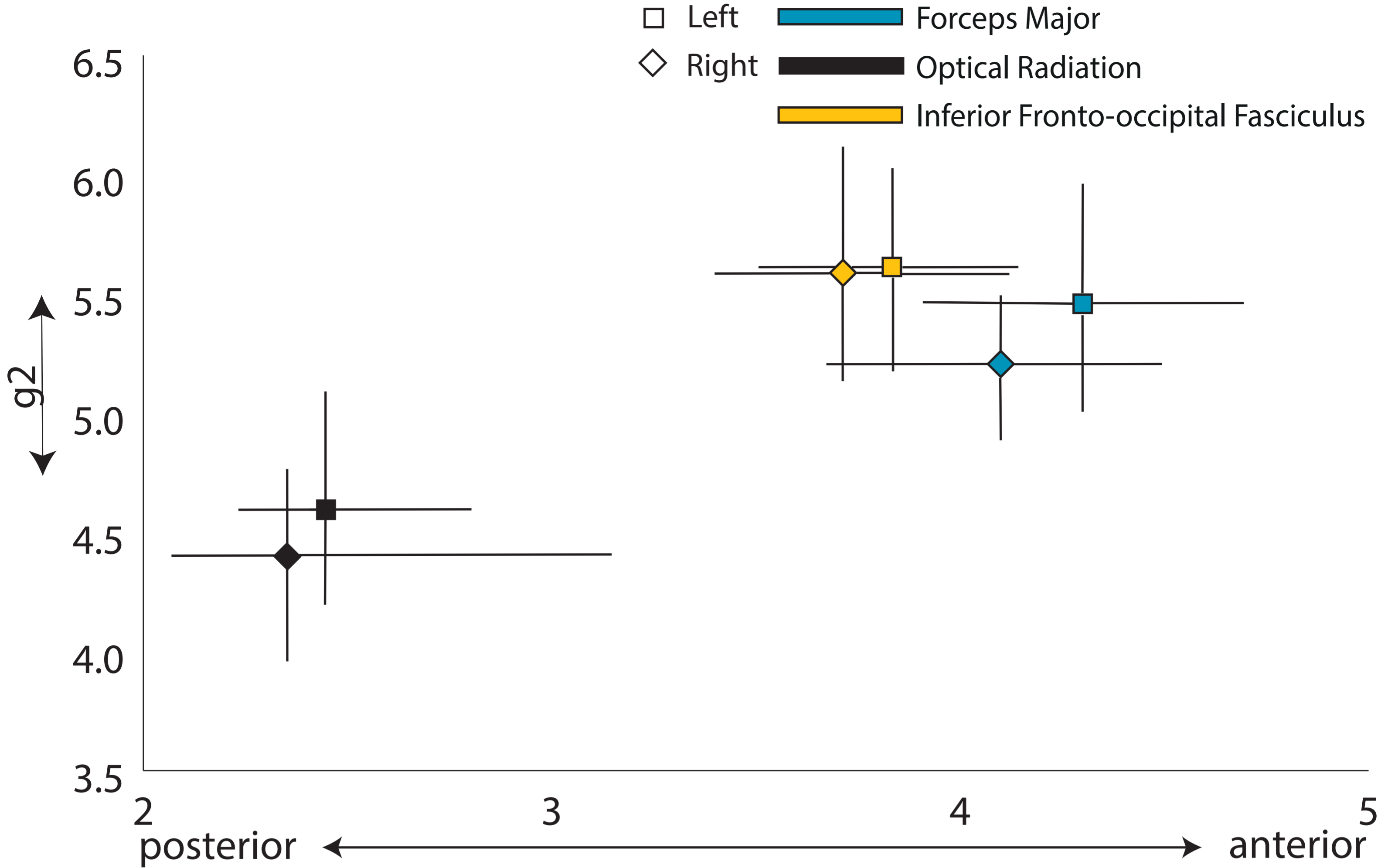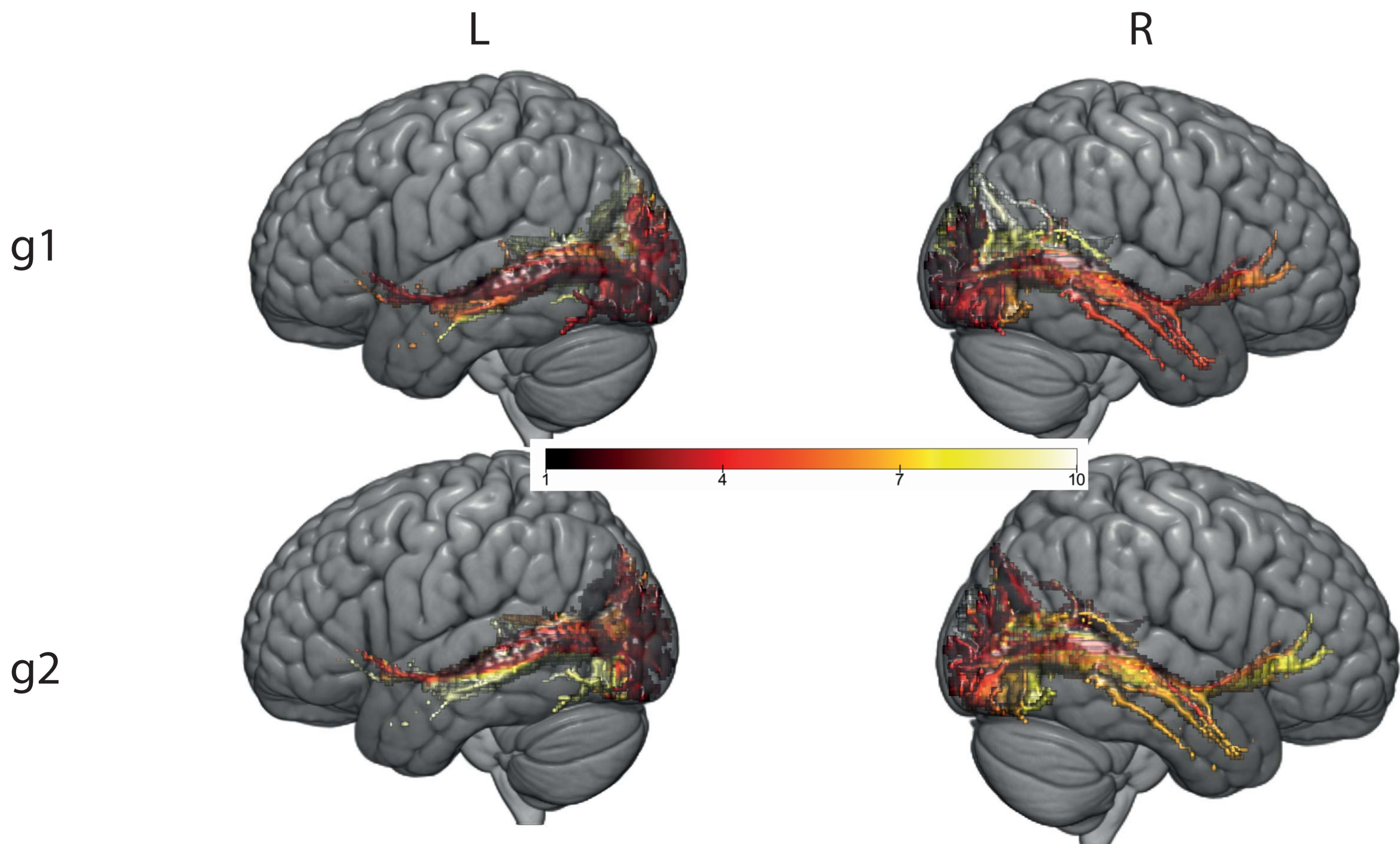

Supplement: Supplementary file 6 — FIGURE S6 (Top) Average value of V1's examined white matter tract projections. Error bars represent the bootstrapped 95% confidence interval of the mean. X axis—Value along the projection image of the dominant connectivity mode (g1). For clarity of interpretation, the direction of the corresponding gradient is indicated under the axis; Y axis—Value along the projection image the second dominant connectivity mode (g2). For clarity of interpretation, the direction of the corresponding gradient is indicated to the left of the axis. (Bottom) Projection images for a representative subject. L and R denote left and right hemispheres, respectively. [file HBM-42-5827-s005.pdf]

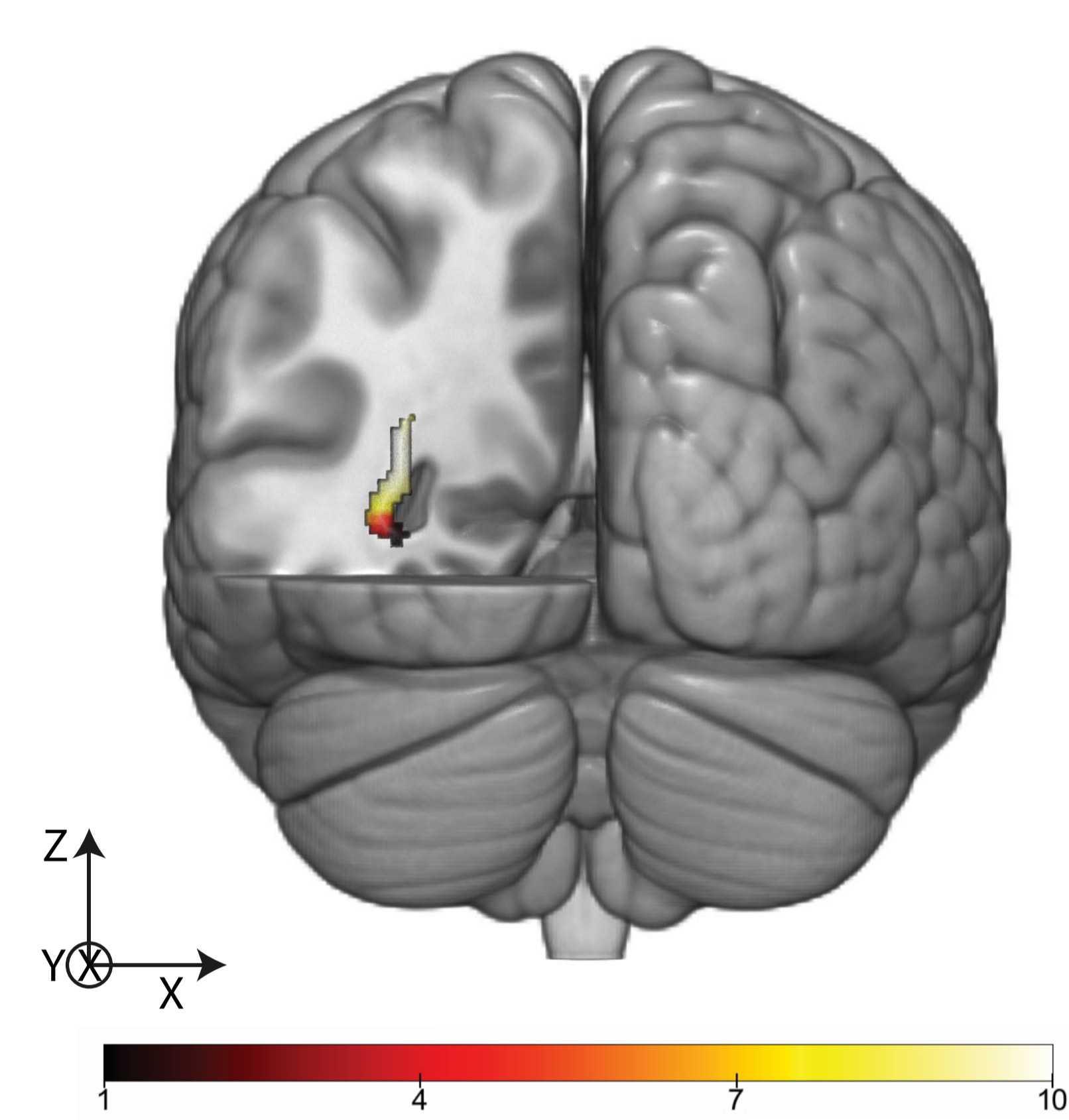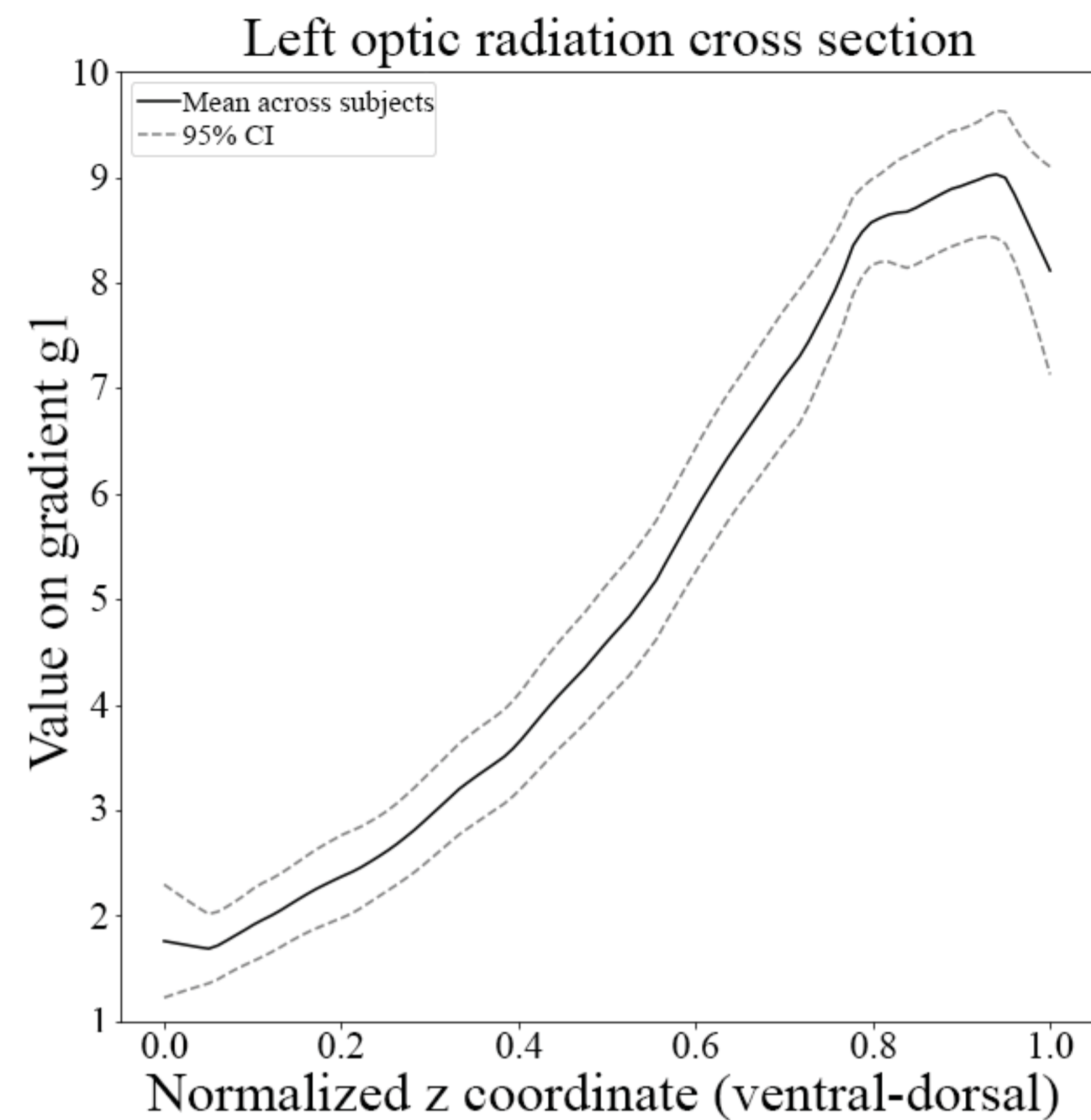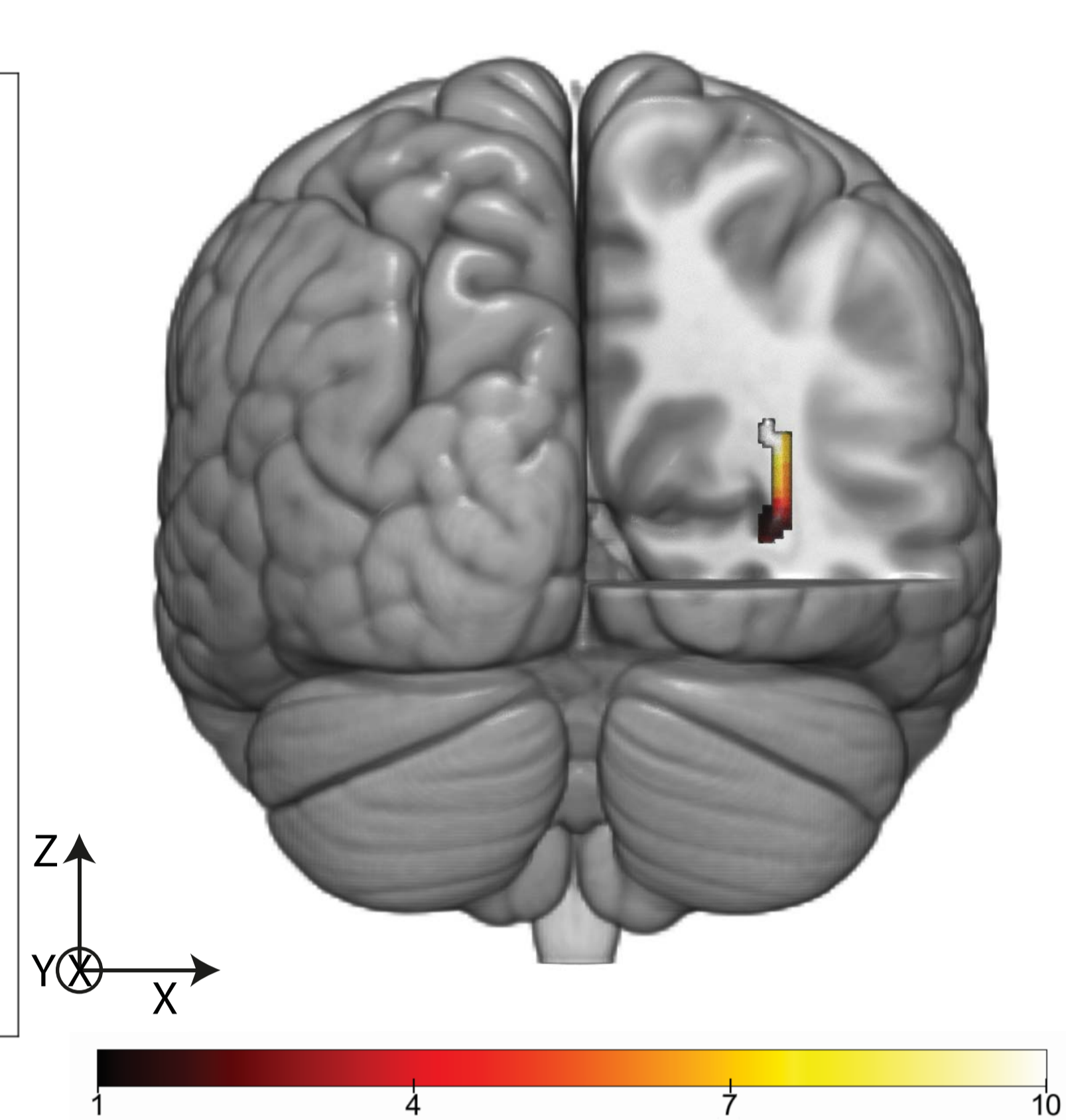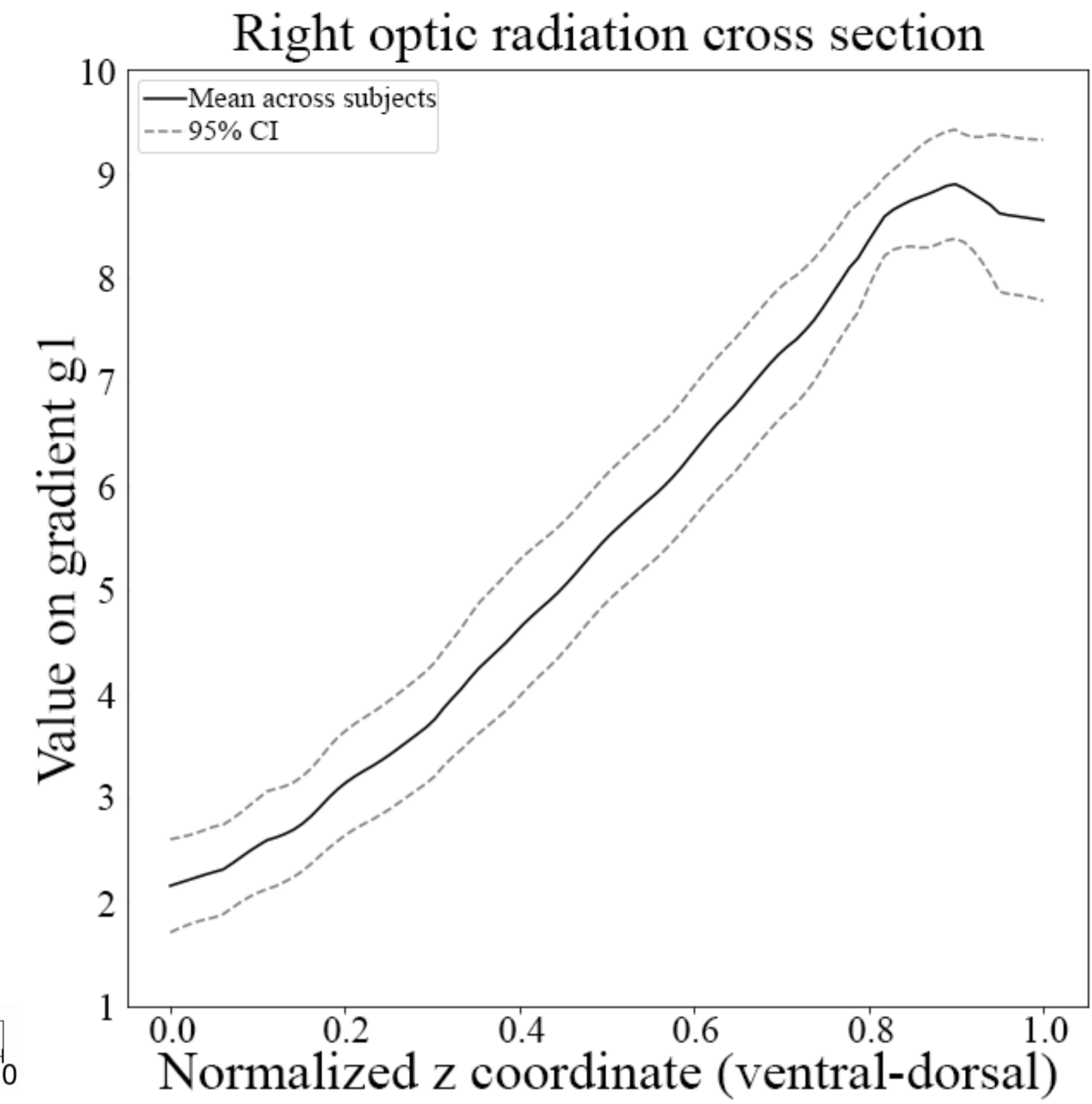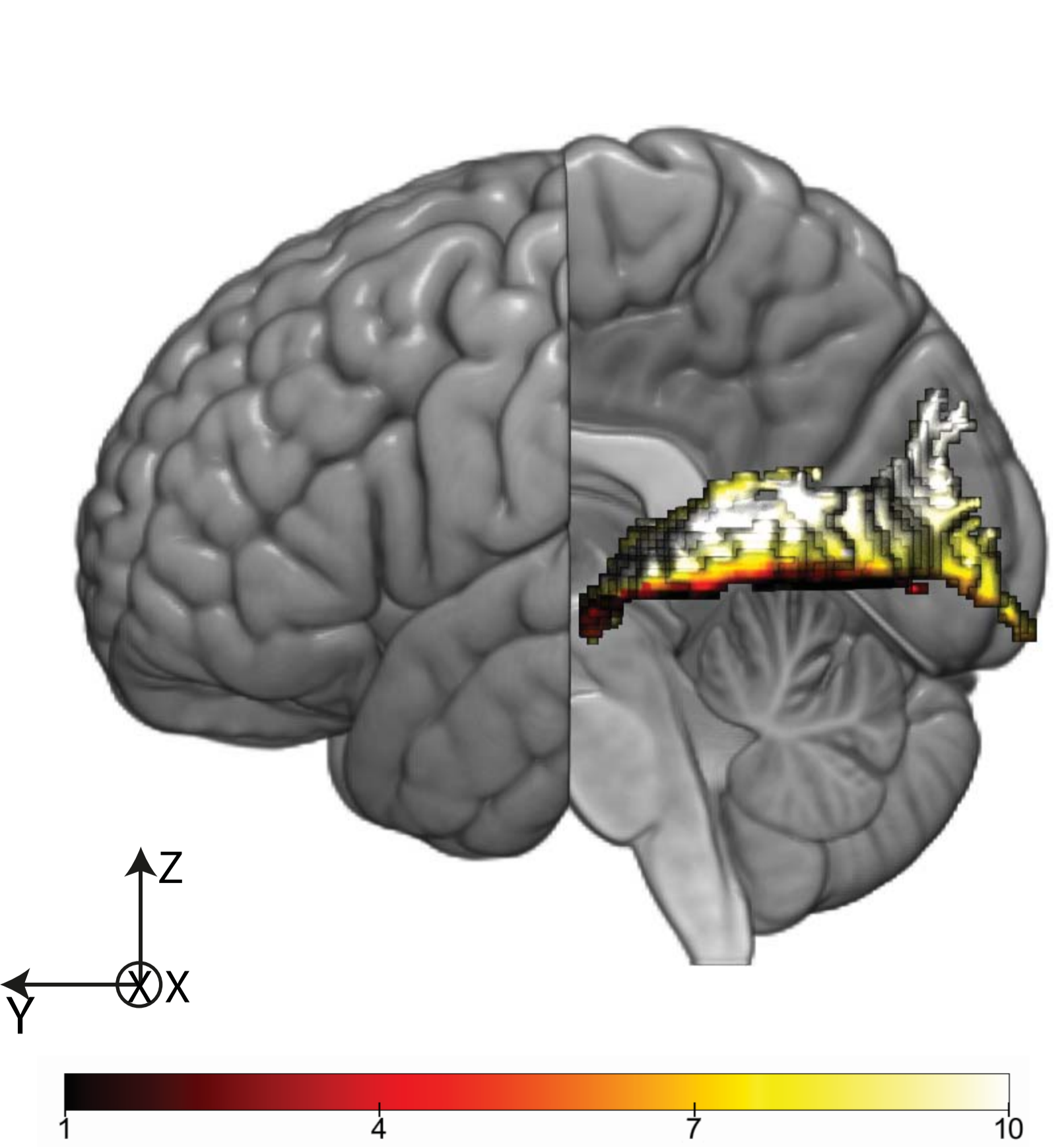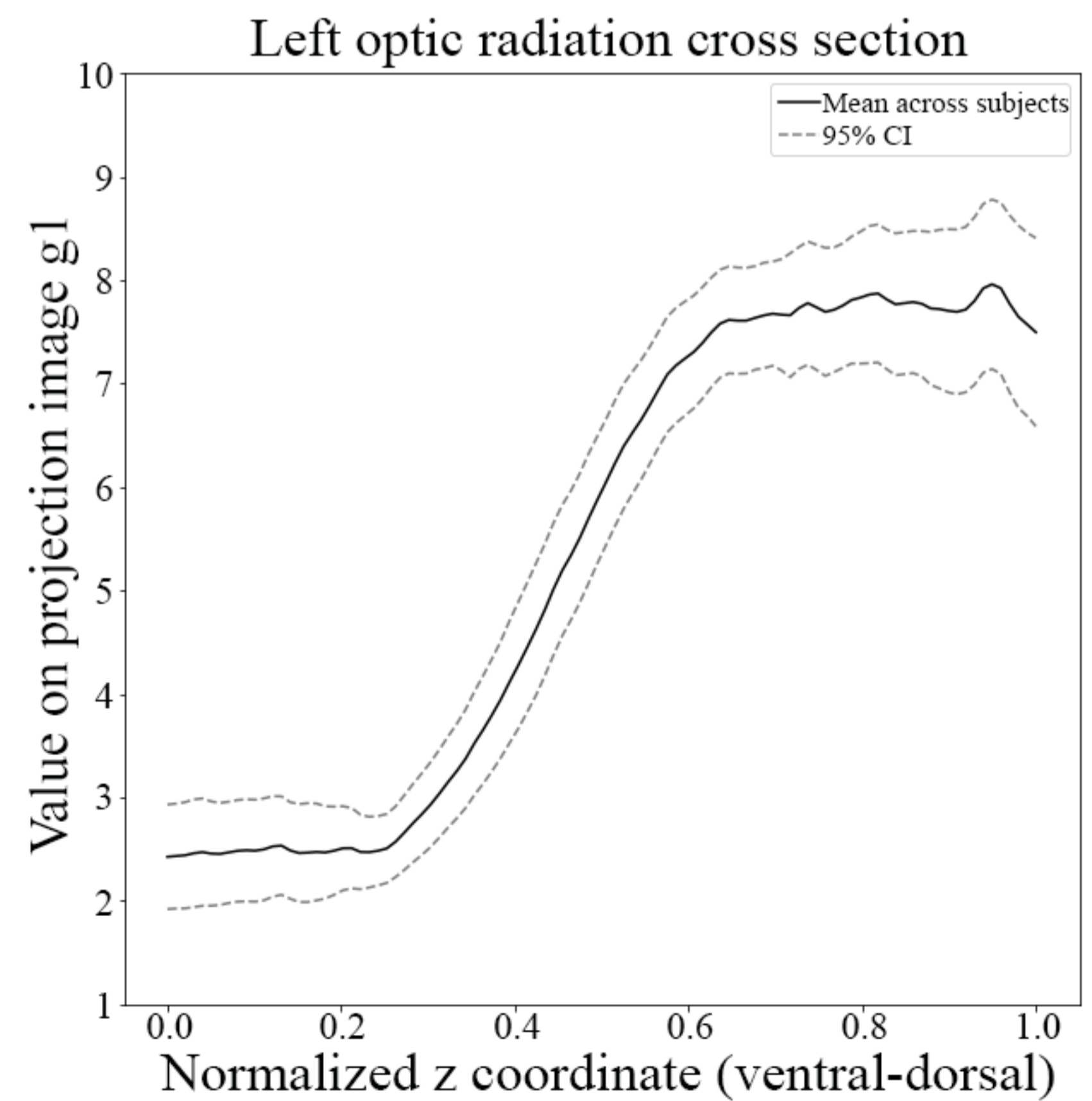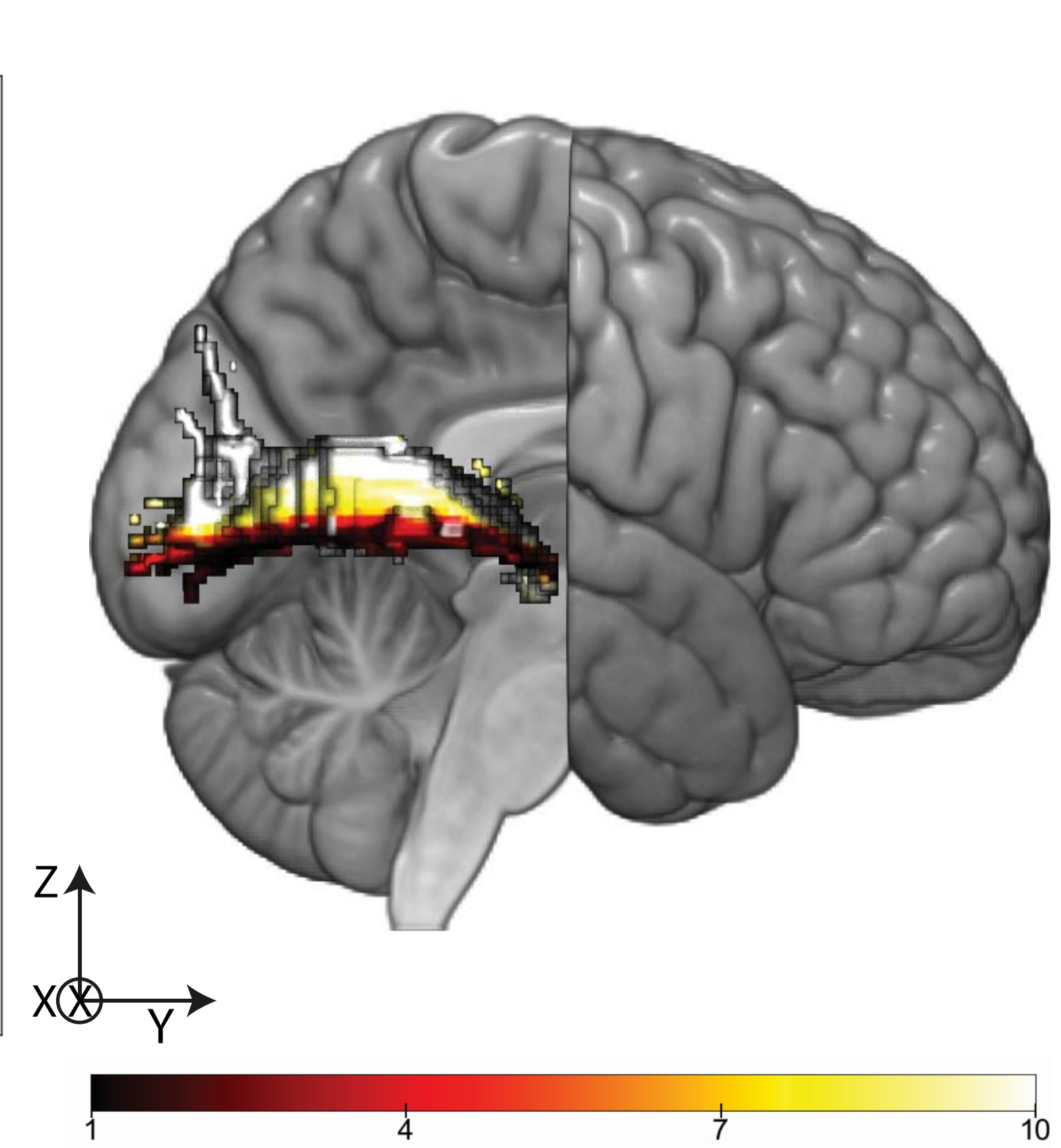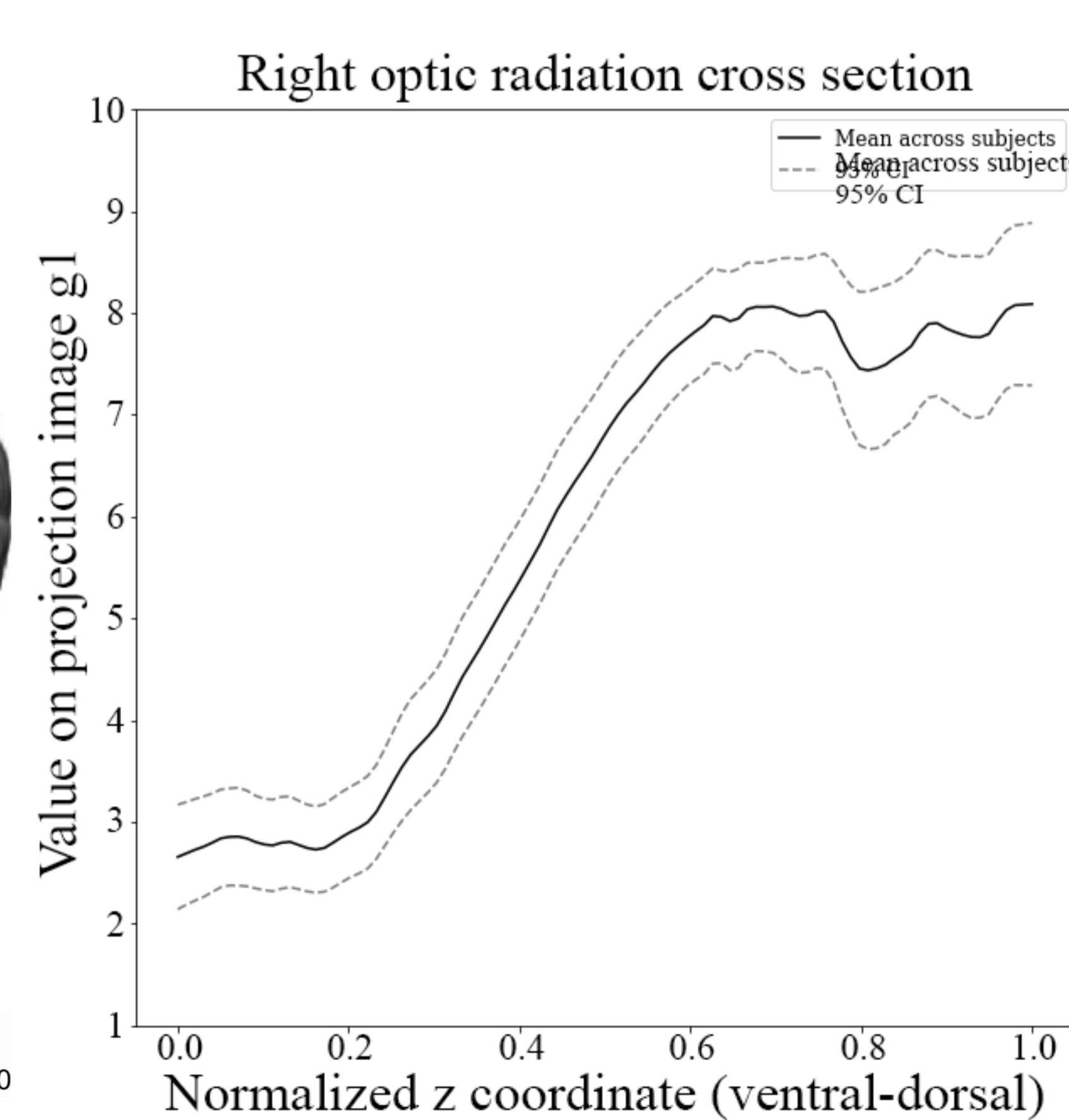

Supplement: Supplementary file 7 — FIGURE S7 TOP—(Left) Representative subjects' left seed gradient cross section (Graph) mean value of gradient g1 (dominant connectivity mode) along the z‐axis on the left optic radiation cross section. (Right) Representative subjects' right seed gradient cross section (Graph) Mean value of gradient g1 (dominant connectivity mode) along the z‐axis on the right optic radiation cross section. Gradient values are normalized between 1 and 10 and the normalized z coordinate represents the range of coordinates of each subject's optic radiation cross section up sampled to 100 data points. The dashed line represents the 95% confidence interval. Bottom—(Left) Representative subjects' left projection image cross section (Graph) mean value of projection image g1 (projected dominant connectivity mode values to the target space) along the z‐axis on the left optic radiation.(Right) Representative subjects' right projection image cross section (Graph) Mean value of projection image g1 (projected dominant connectivity mode values to the target space) along the z‐axis on the right optic radiation. Projection image values are normalized between 1 and 10 and the normalized z coordinate represents the range of coordinates of each subject's optic radiation up sampled to 100 data points. The dashed line represents the 95% confidence interval [file HBM-42-5827-s001.pdf]

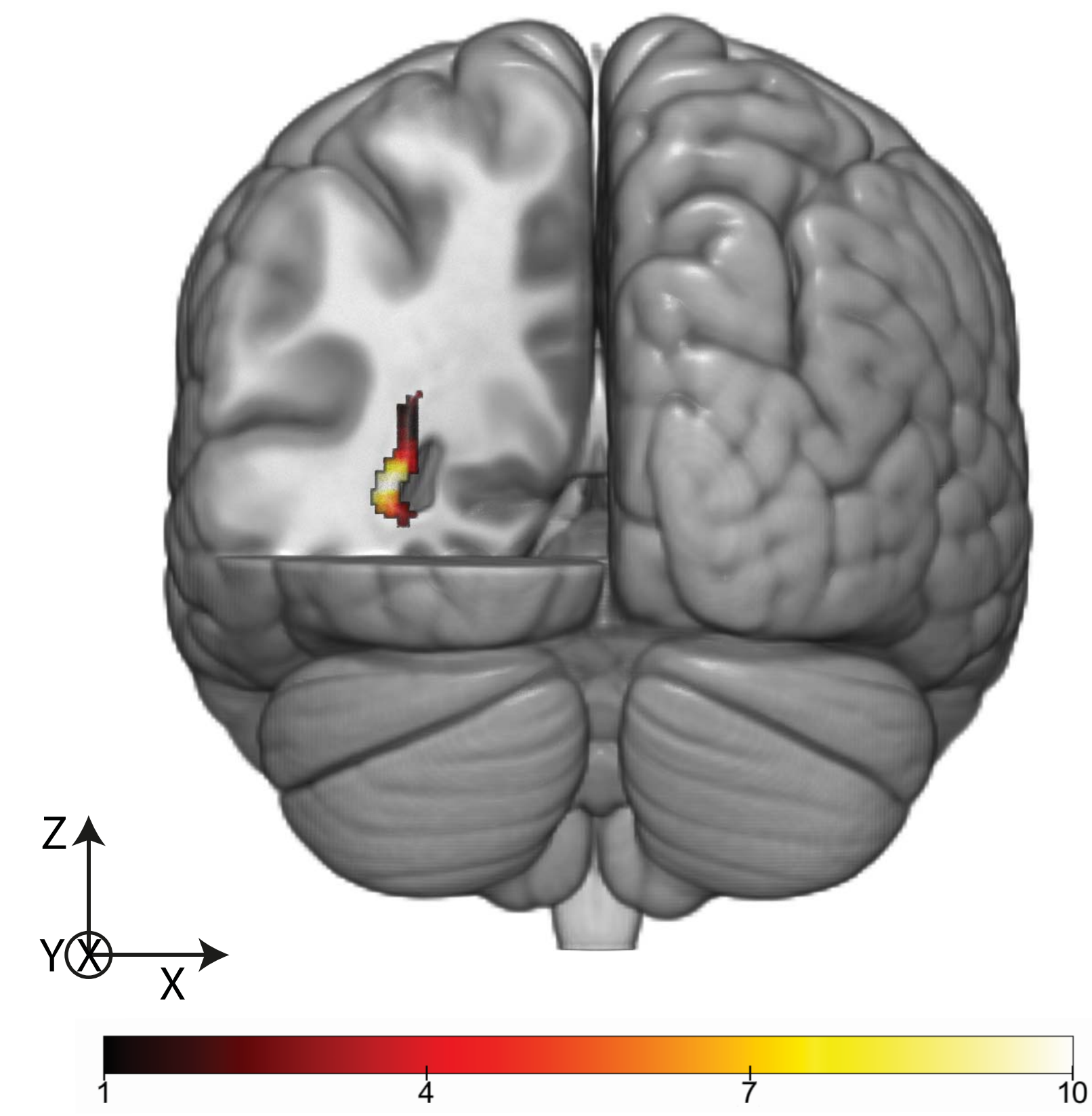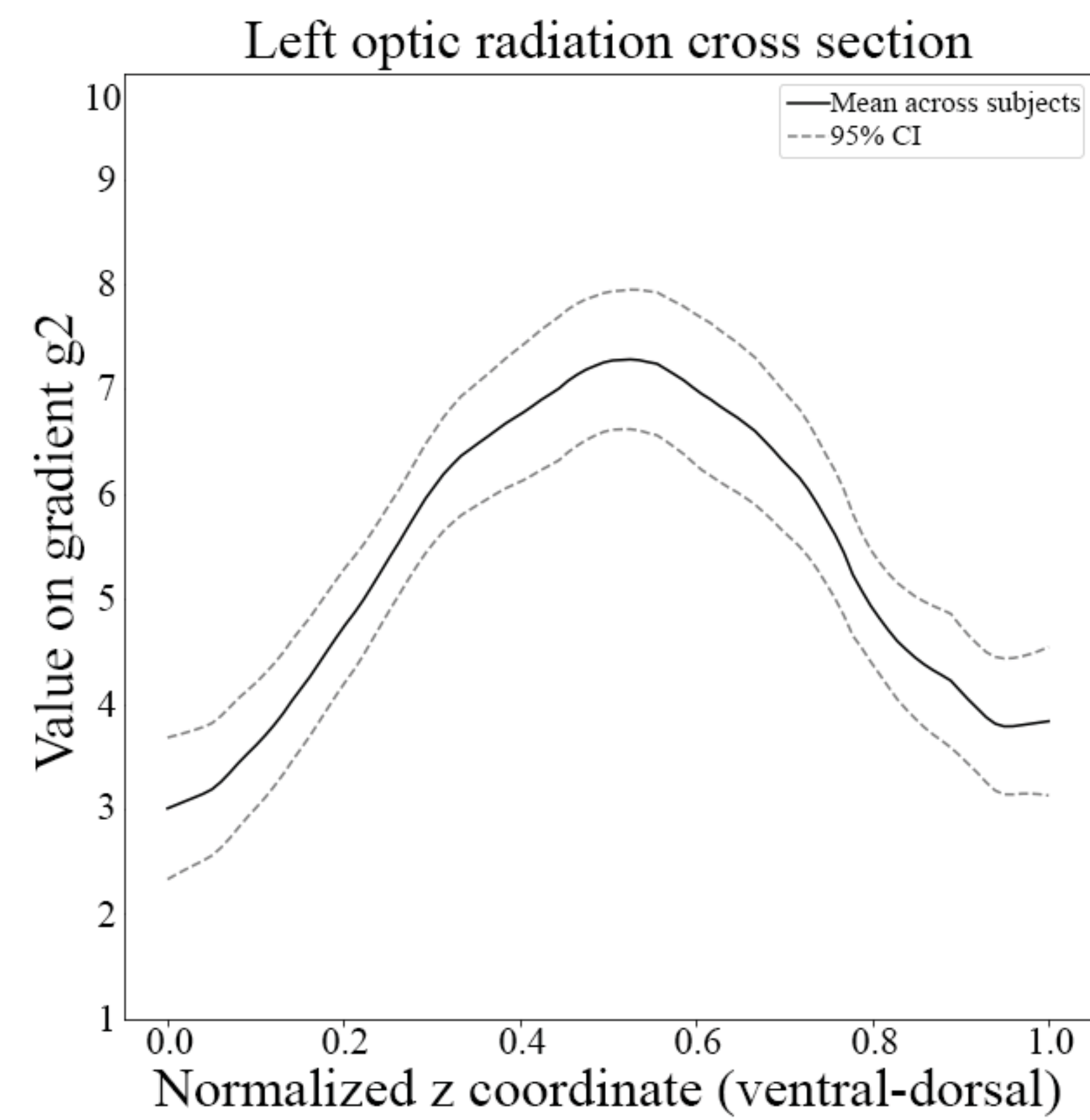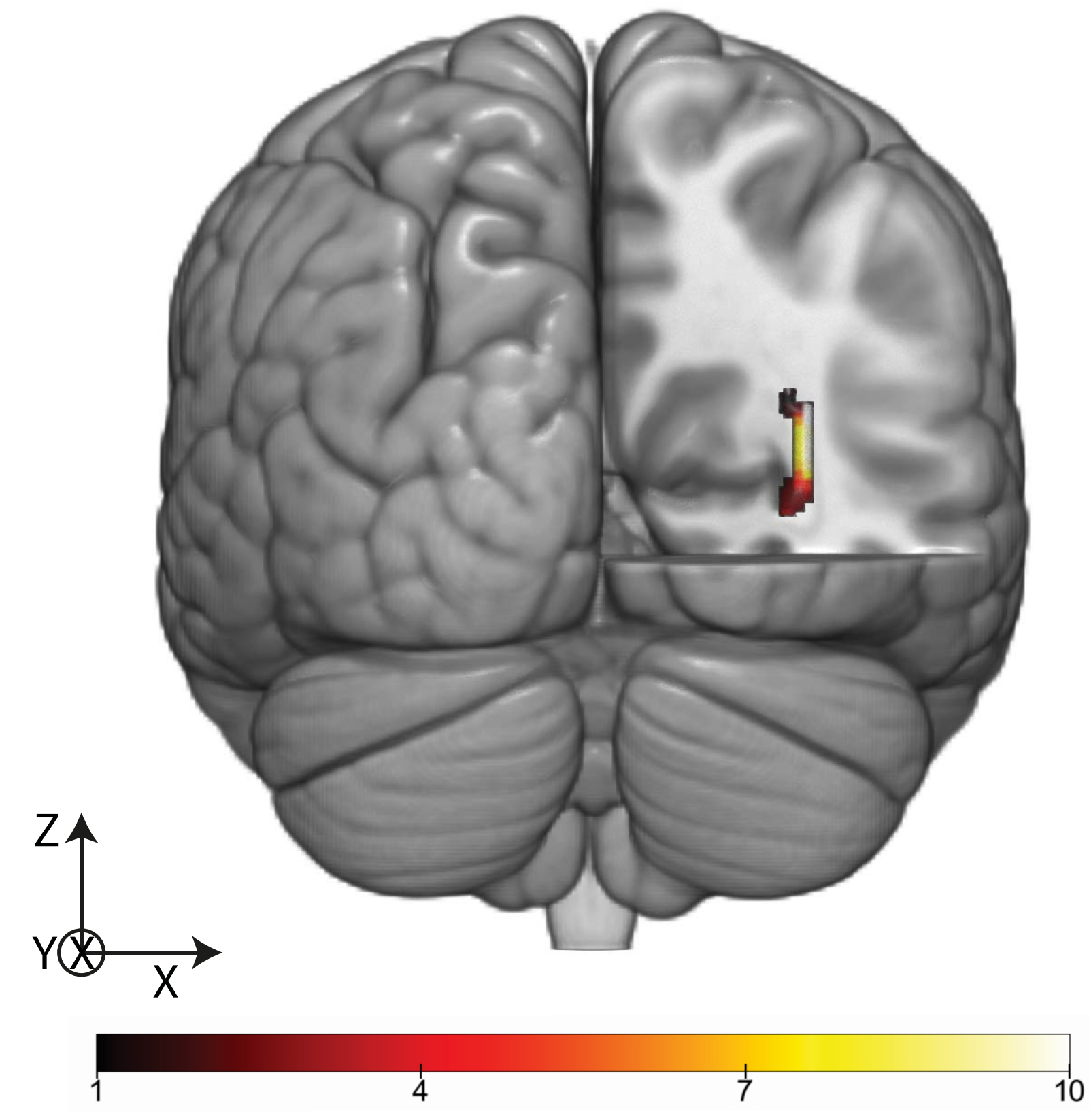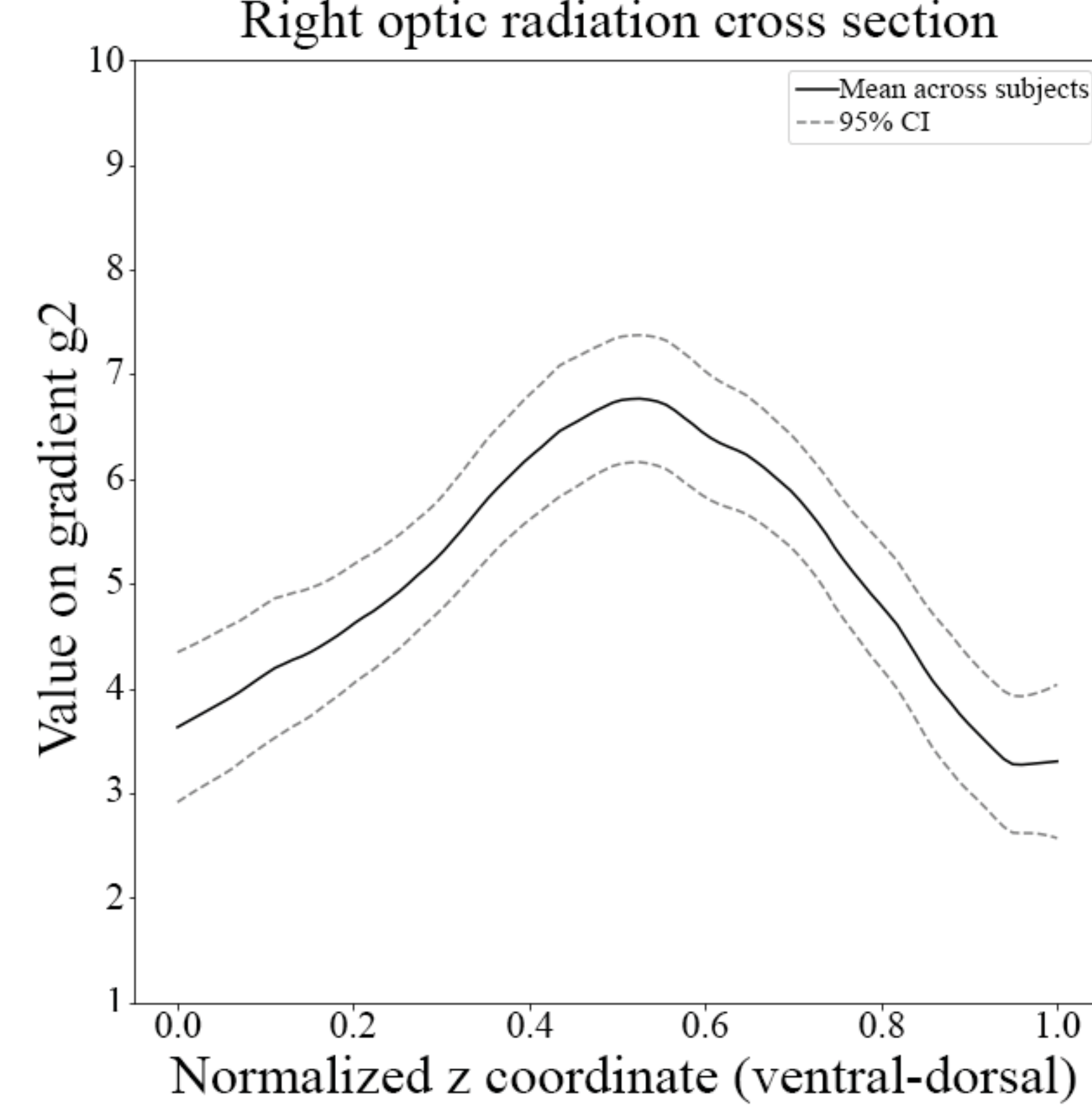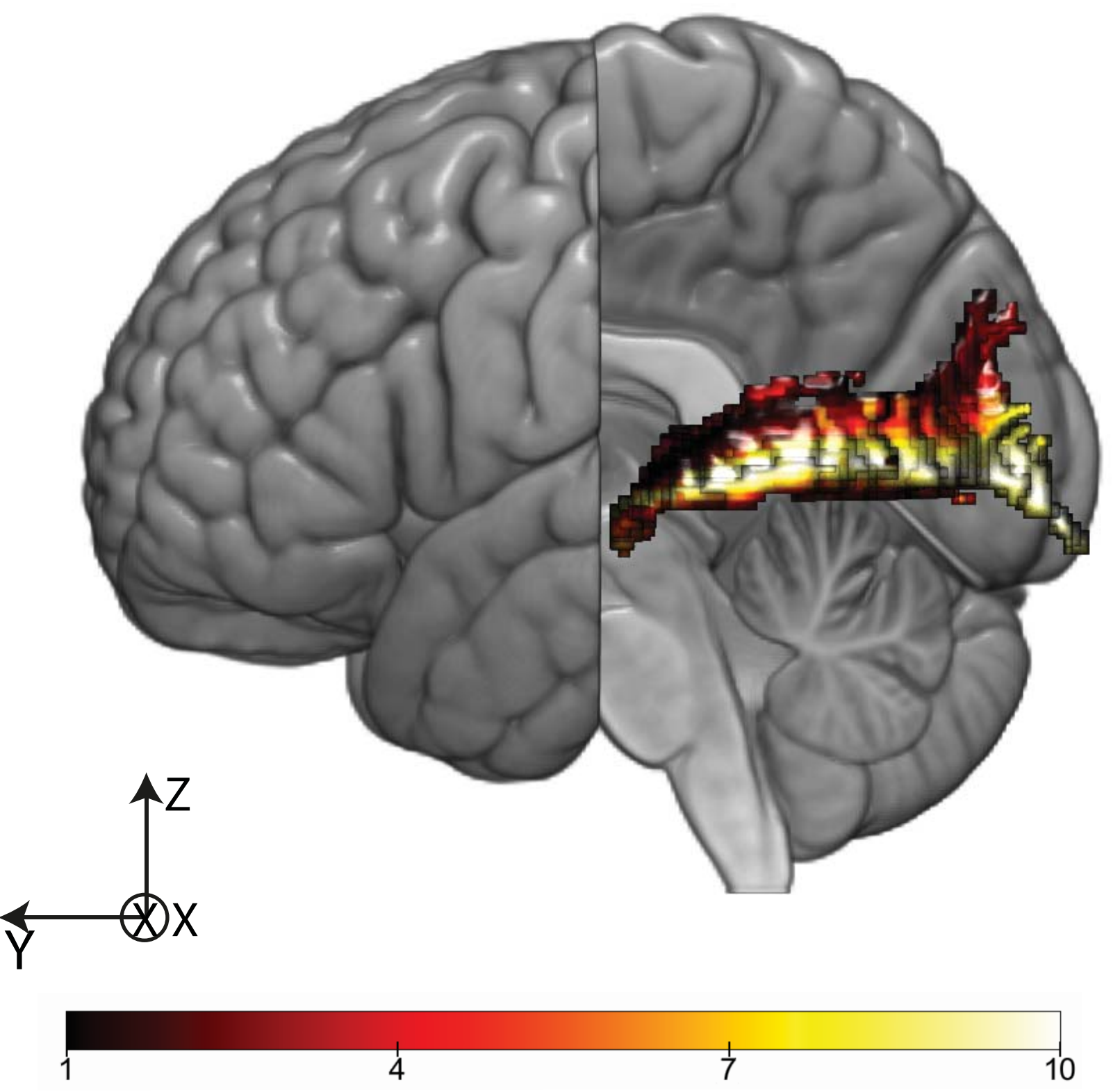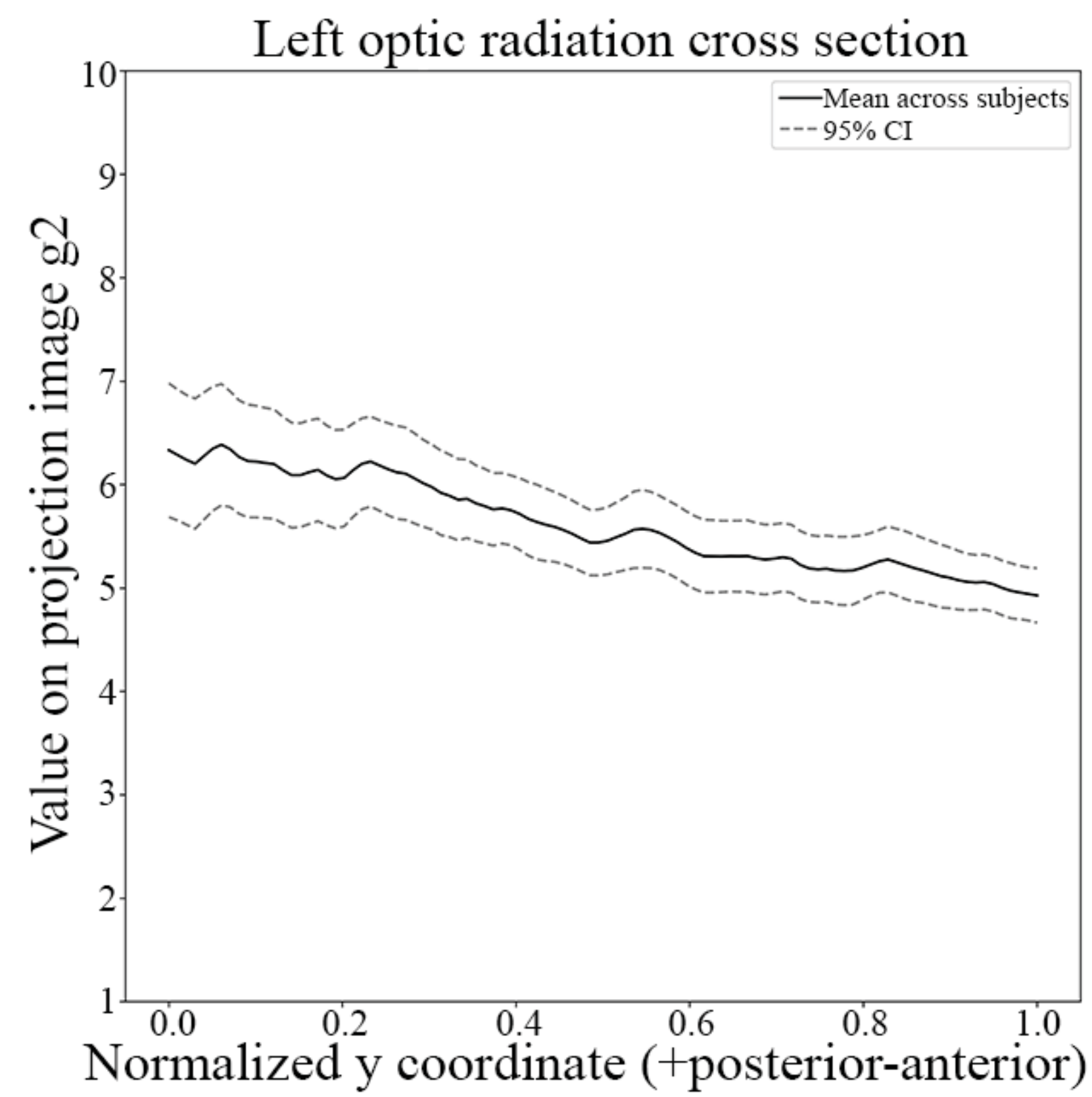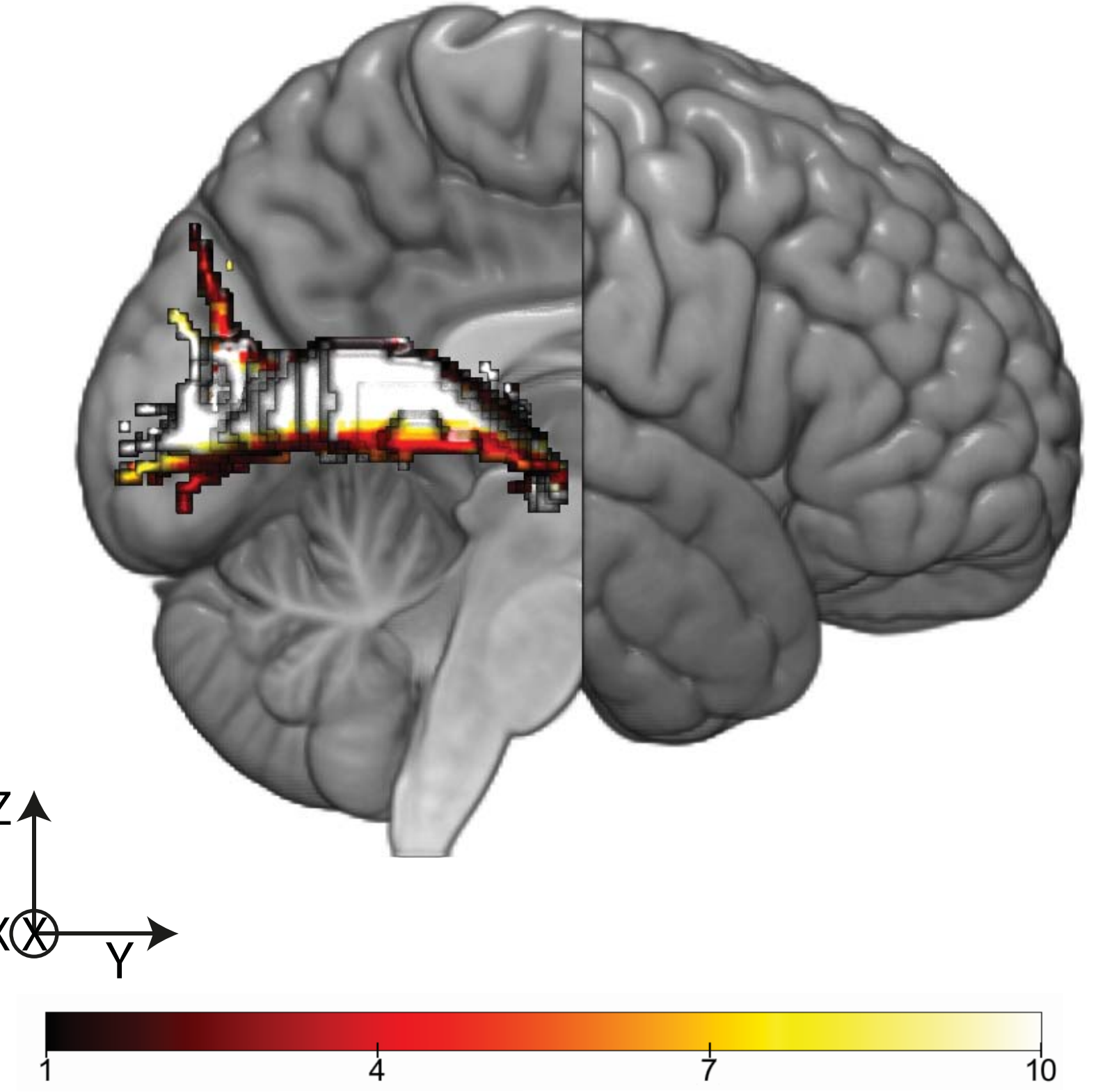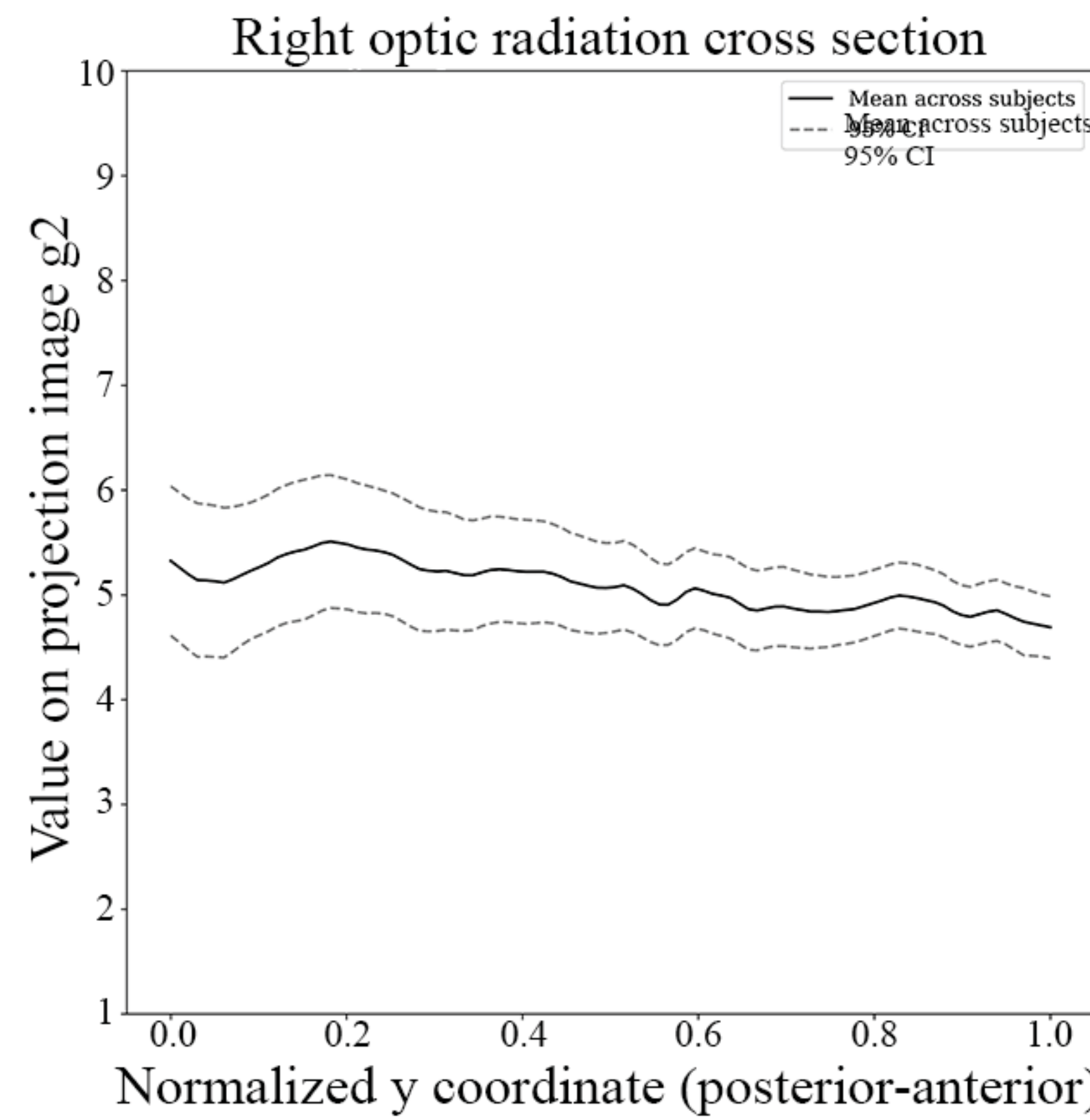

Supplement: Supplementary file 8 — FIGURE S8 TOP—(Left) Representative subjects' left seed gradient cross section (Graph) Mean value of gradient g2 (second dominant connectivity mode) along the z‐axis on the left optic radiation cross section. (Right) Representative subjects' right seed gradient cross section (Graph) mean value of gradient g2 (second dominant connectivity mode) along the z axis on the right optic radiation cross section. Gradient values are normalized between 1 and 10 and the normalized z coordinate represents the range of coordinates of each subject's optic radiation cross section up sampled to 100 data points. The dashed line represents the 95% confidence interval. BOTTOM ‐ (Left) Representative subjects' left projection image cross section (Graph) Mean value of projection image g2 (projected second dominant connectivity mode values to the target space) along the z‐axis on the left optic radiation.(Right) Representative subjects' right projection image cross section (Graph) mean value of projection image g2 (projected second dominant connectivity mode values to the target space) along the z axis on the right optic radiation. Projection image values are normalized between 1 and 10 and the normalized z coordinate represents the range of coordinates of each subject's optic radiation up sampled to 100 data points. The dashed line represents the 95% confidence interval. [file HBM-42-5827-s002.pdf]
